# Convergent Evolution at the Gametophytic Self-Incompatibility System in *Malus* and *Prunus*

**DOI:** 10.1371/journal.pone.0126138

**Published:** 2015-05-19

**Authors:** Bruno Aguiar, Jorge Vieira, Ana E. Cunha, Nuno A. Fonseca, Amy Iezzoni, Steve van Nocker, Cristina P. Vieira

**Affiliations:** 1 Instituto de Investigação e Inovação em Saúde, Universidade do Porto, Porto, Portugal; 2 Instituto de Biologia Molecular e Celular (IBMC), Universidade do Porto, Porto, Portugal; 3 CRACS-INESC Porto, Rua do Campo Alegre 1021/1055, 4169–007, Porto, Portugal; 4 European Bioinformatics Institute (EMBL-EBI), Welcome Trust Genome Campus, CB10 1SD, Cambridge, United Kingdom; 5 Michigan State University, East Lansing, Michigan, United States of America; Chiba University, JAPAN

## Abstract

*S-RNase*-based gametophytic self-incompatibility (GSI) has evolved once before the split of the Asteridae and Rosidae. This conclusion is based on the phylogenetic history of the *S-RNase* that determines pistil specificity. In Rosaceae, molecular characterizations of *Prunus* species, and species from the tribe Pyreae (*i*.*e*., *Malus*, *Pyrus*, *Sorbus*) revealed different numbers of genes determining *S*-pollen specificity. In *Prunus* only one pistil and pollen gene determine GSI, while in Pyreae there is one pistil but multiple pollen genes, implying different specificity recognition mechanisms. It is thus conceivable that within Rosaceae the genes involved in GSI in the two lineages are not orthologous but possibly paralogous. To address this hypothesis we characterised the *S-RNase* lineage and *S*-pollen lineage genes present in the genomes of five Rosaceae species from three genera: *M*. *× domestica* (apple, self-incompatible (SI); tribe Pyreae), *P*. *persica* (peach, self-compatible (SC); Amygdaleae), *P*. *mume* (mei, SI; Amygdaleae), *Fragaria vesca* (strawberry, SC; Potentilleae), and *F*. *nipponica* (mori-ichigo, SI; Potentilleae). Phylogenetic analyses revealed that the *Malus* and *Prunus S-RNase* and *S*-pollen genes belong to distinct gene lineages, and that only *Prunus S-RNase* and *SFB*-lineage genes are present in *Fragaria*. Thus, *S-RNase* based GSI system of *Malus* evolved independently from the ancestral system of Rosaceae. Using expression patterns based on RNA-seq data, the ancestral *S-RNase* lineage gene is inferred to be expressed in pistils only, while the ancestral *S*-pollen lineage gene is inferred to be expressed in tissues other than pollen.

## Introduction

Self-incompatibility (SI), a genetic barrier to self-fertilisation in which the female reproductive cells discriminate between genetically related and non-related pollen, and reject the former [1], has evolved at least 35 times independently in different flowering plant lineages (see for instance Igic et al. [2]). *RNase* based gametophytic SI (GSI), a system where pollen tube growth that expresses a specificity that matches either of those expressed in the style is inhibited by a pistil ribonuclease, called *S-RNase*, is present in core-eudicots such as Solanaceae, Plantaginaceae, Rubiaceae (all Asteridae plant families), and Rosaceae (Rosidae) [3,4]. According to phylogenetic analyses of the *T2-RNase* gene family, this system has the peculiarity of a single evolution that predates the split of the Asteridae and Rosidae, about 120 million years ago [5–7]. When *RNase* based GSI is lost, the system is never regained [2,8–10].

Comparative analyses have been performed, using divergent species, to understand how the system has evolved. In this system there is evolutionary plasticity. For instance, within Rosaceae, pollen specificity is determined by one F-box gene in *Prunus* (called *SFB*, *S*-haplotype specific F-box gene; [11–18], but multiple genes determine Pyreae (*Malus*, *Pyrus*, and *Sorbus*) pollen specificity (called *SFBB*s, *S*-locus F-box brothers genes; [19–23]. It should be noted that, in both cases, mutations at the *S*-pistil and *S*-pollen genes lead to loss of specificity recognition [24–27], thus supporting the correct identification of the genes determining GSI specificity. The multiplicity of pollen genes determining GSI is also observed in Solanaceae, both in *Petunia*, and *Nicotiana* (the genes are called *SLFs*, *S*-locus F-box; [28,29]).

The different numbers of *S*-pollen genes implies different mechanisms of self-pollen recognition in Pyreae and *Prunus* [16,29–32]. Although Pyreae and *Prunus* species have been diverging for about 32 MY [33], the phylogenetic relationship of *S*-pollen genes from the two species groups is still unclear [19,28,34–36]. Nevertheless, in phylogenetic analyses *Malus SFBB* genes always cluster with *Prunus SLFL*s (*S*-locus F-box like) genes, that are the closest neighbour genes of the *Prunus S*-locus [12,13]. There is, however, no evidence for these genes being involved in pollen GSI specificity since they are expressed in tissues other than pollen and anthers [12,13], and their levels of diversity are markedly lower than those at the *Prunus S*-locus genes [7,18,37] or the *Malus SFBB* genes [22,23]. Furthermore, the deletion of the *SLFL1* gene in *P*. *avium S3*- haplotype does not affect GSI specificity recognition [38]. *Prunus SFB* sequences, on the other hand, depending on the settings and alignment algorithm used in phylogenetic analyses, are shown as a very divergent group, or alternatively, as a sister group to a group of sequences that include the *Petunia*, *Antirrhinum* and Pyreae *S*-pollen genes [19,28,34–36].

Evolution by gene duplication has been suggested to explain the evolution of the *S*-pollen genes in Rosaceae [28,34], but has never been explicitly addressed. It should be noted that a single evolution of the *S-RNase* gene does not exclude the possibility that *S-RNase* paralogs could be determining pistil specificity in different species. For instance in the Brassicaceae family, that exhibits sporophytic SI (SSI), in both *Arabidopsis* and *Brassica* genera the female component is a transmembrane receptor kinase (the *SRK* gene) and the male component is a cysteine rich gene (the *SCR* gene), but in *Leavenworthia* the *S*-locus genes have secondarily evolved from paralogs of *SRK* and *SCR* [39]. Thus, within Brassicaceae, the *S*-locus genes evolved twice independently, although the system works in a similar way in the three genera [40–42]. It is thus, possible that in Rosaceae, the differences observed are due to a secondarily evolution from paralogs in one of the lineages. Determining whether this is the case, is crucial to the molecular characterization of the *S*-locus region in other species presenting the GSI system.

Genomes are now available for species of three Rosaceae genera, namely *Malus × domestica* (SI; [43]), *Prunus persica* (SC; [44]), *P*. *mume* (SI; [45]), *Fragaria vesca* (SC; [46]) and *F*. *nipponica* (SI; [47]). The complete set of *S-RNase* lineage genes, and genes showing homology to *S*-pollen genes, coupled with synteny information can give insight into the evolution of GSI in Rosaceae. *Fragaria* shares the most common ancestor with *Prunus* and *Malus* lineages 62 MY ago [33,48]. The molecular characterization of the *Fragaria S*-locus has not been performed, but there is evidence for the involvement of stylar *RNases*, although other loci may be involved as well [49]. Analyses of the ribonuclease zymograms of the seedling of the F1, F2 and back crossing of crosses between SC and a SI *Fragaria* species, led to the identification of two unlinked *RNase* loci, one located in chromosome 1 (called *S*-locus) and the other in chromosome 6 (called *T*-locus) [49]. In this system a single active allele at either of the two loci is sufficient to confer self-incompatibility. Thus, self-compatibility implies being homozygous for null alleles at both loci [49]. Such inference depends, however, on the existence of the null alleles at each locus. These null alleles are associated with a lack of *RNase* activity. Nevertheless, because of the methodology used (isoelectric focusing of the stylar native protein extract and staining for ribonuclease activity), the different number of ribonuclease proteins in the different species may reflect differences in other ribonucleases not involved in GSI. Indeed, ribonucleases not determining GSI can also be expressed in pistil tissues [50].

In this work we test the hypothesis of evolution from paralogs for the *Prunus* and *Malus* GSI genes, by performing phylogenetic analyses of the *Malus*, *Prunus* and *Fragaria T2-RNas*e and *SFBB*-*SFB* genes identified in five Rosaceae genomes. We also use macro and micro synteny data to support our findings. *T2 RNase* and F-box expression patterns were also characterized in order to understand how difficult it is to evolve the restricted expression pattern shown by *S*-pistil and *S*-pollen genes. Inferences on the *Fragaria* and ancestral Rosaceae *S*-locus are presented.

## Methods

### Identification of *M*. *× domestica*, *P*. *persica*, *P*. *mume*, *F*. *vesca*, and *F*. *nipponica S-RNase*, *S-RNase*-like genes, *SFB*, *SLFL*, and *SFBB*—like genes

*S*-*RNase* like genes in the *M*. × *domestica* (a SI species), genome were identified by homology with the *M*. × *domestica* S2-RNase (AAA79841.1), using blastp (Expect value (e)<0.05) and the predicted peptides Database from *M*. × *domestica* Whole Genome v1.0 Assembly (http://www.rosaceae.org; [43]). A similar approach was used for *P*. *persica* (SC), using peach S1-RNase (BAF42768.1) as the query and the Peach genome v1.0 predicted peptides Database (http://www.rosaceae.org; [44]), and for *Fragaria vesca* (SC), using *M*. *× domestica* S4-RNase (AF327223.1) and *P*. *avium* S23-RNase (AY259114.1) as queries, and the *F*. *vesca* Genome v1.0 ab initio gene proteins (http://www.rosaceae.org; [46]). Non-annotated *S-RNase* genes and potential *S-RNase* lineage pseudogenes in these genome drafts, were identified by homology using local tblastn with the above query sequences, and putative open reading frames longer than 100 bp (getorf; http://emboss.sourceforge.net; [51]). For *P*. *mume* (SI; http://prunusmumegenome.bjfu.edu.cn; [45]), *S-RNase* like genes were identified by homology using local tblastn with the above query sequences, and putative open reading frames longer than 100 bp (getorf; http://emboss.sourceforge.net; [51]). For *F*. *nipponica* [47]) we used NCBI blastn (word size of 7 and standard algorithm parameters) using as query the above sequences and as database *F*. *nipponica* whole-genome shotgun contigs (wgs). It should be noted that the *F nipponica* genome assembly is not available. In some cases, sequences were curated by introduction of sequence gaps to extend recognizable homology with the query sequence. The presence of amino acid pattern 4 ([CG]P[QLRSTIK][DGIKNPSTVY]) that is absent in *S-RNases* and *S*- lineage genes (genes similar to functional *S-RNases* but not involved in GSI; [7]), but present in other *T2- RNases* [4,7] was recorded for each sequence. Because *S-RNases* present at maximum two introns [5,52], the number of putative introns was determined for these sequences. Since the isoelectric point of the *S-RNase* proteins varies between 8 and 10 [3], for all peptides, isoelectric points were calculated using software available through ExPASy [53].

*SFB*, *SLFL* and *SFBB*—like genes were identified as described above using *M*.*× domestica* SFBB3-beta (BAF47180.1) and *P*. *avium* SFB3 (AAT72121.1) as queries for *M*. × *domestica*, SFBB3-beta, *P*. *avium* SLFL1 (BAG12295.1) and SFB3 as queries for *P*. *persica* and *P*. *mume*, and SFBB3-beta and SFB3 as queries for *F*. *vesca* and *F*. *nipponica*. Query sequences were trimmed to eliminate the F-box region, and only matches with e<E-12 were considered. For the *M*. *× domestica*, *P*. *persica* and *F*. *vesca* genes here obtained, the genomic location was obtained using GBROWSE at GDR (http://www.rosaceae.org; [43]) to retrieve the sequence of the region and blast two sequences (bl2seq) to obtain the location of the gene sequence used.

### Phylogenetic analyses

Phylogenetic analyses utilized Muscle, ClustalW2, and T-Coffee alignment algorithms as implemented in ADOPS [54]. It should be noted that when ADOPS is used, nucleotide sequences are first translated and then aligned using the amino acid alignment as a guide. Only codons with a support value above 2 were used for phylogenetic reconstruction. Bayesian trees were obtained using MrBayes 3.1.2 [55], as implemented in the ADOPS pipeline. The Generalised Time-Reversible (GTR) model of sequence evolution was implemented in the analyses, allowing for among-site rate variation and a proportion of invariable sites. Third codon positions were allowed to have a gamma distribution shape parameter different from that of first and second codon positions. Two independent runs of 2,000,000 generations with four chains each (one cold and three heated) were carried out. The average standard deviation of split frequencies was always ~0.01 and the potential scale reduction factor for every parameter was ~1.00, showing that convergence was achieved. Trees were sampled every 100th generation and the first 5000 samples were discarded (burn-in). The remaining trees were used to compute the Bayesian posterior probabilities for each clade of the consensus tree.

### Expression analyses of *Malus S-RNase*, *S-RNase* lineage, *SFB*, *SFBB*, *and SFBB-* like genes

To estimate expression of the *Malus S- RNase* like and *SFB*-like genes, we use RNA-seq data from *M*. *fusca* (a diploid wild apple species; http://vannocke.hrt.msu.edu/DBI-0922447/RNA-seq.html; S. van Nocker, manuscript in preparation; S1 Table). Before assembly, adaptor sequences were removed from raw reads. FASTQC reports were then generated and based on this information the resulting reads were trimmed at both ends. Nucleotide positions with a score lower than 20 were masked (replaced by an N). These analyses were performed using the FASTQ tools implemented in the Galaxy platform [56–58]. The resulting high-quality reads were used in the subsequent transcriptome assembly using Trinity with default parameters [59]. FPKM values were estimated using the eXpress software [60] as implemented in Trinity. All contigs were used as queries for tblastn searches using local blast [61]. The query sequences used, except for the *S*-locus genes, were those of *M*. *domestica* (*RNase S*-lineage 1 *MDP0000210735A*; *Malus RNase S*-lineage 2 *MDP0000682955*; *Malus SLFL3*-like *MDC010871*; *Malus SLFL*-like *MDP0000266067* and *MDP0000302221*; and *Malus SFB*-like *MDP0000890198* and *MDP0000393954*), since divergence values between *M*. *× domestica* and *M*. *fusca* are low (~0.02 [33,62]). In contrast, divergence values for *S*-locus genes are generally above 0.2 [37]. Therefore, query sequences for the *S-RNase* and *SFBB* genes were first obtained from *M*. *fusca*, whereas query sequences for *S-RNase* like and *SFBB* like genes were those previously annotated in *M*. × *domestica*. For the *S-RNase* gene, *M*. *fusca* genomic DNA and primers SorbusRNaseF (5'..AAGTTGTTTACGGTTCAC..3') and SorbusRNaseR (5'..TATTCTTTTGGCACTTGA..3') were used for PCR with standard amplification conditions [35 cycles of denaturation at 94°C for 30 seconds, primer annealing at 48°C for 30 s, and primer extension at 72°C for 3 min; [23]]. For the *SFBB* gene, *M*. *fusca* genomic DNA and primers SFBBgenF (5'..AAGTCYCTGATGMGRTTC..3') and SFBBgenR (5'..GTCCATTACCCAYRTYTC..3') were used with the same amplification conditions. For each amplification product, four amplicons were sequenced in order to obtain a consensus sequence. The *M*. *fusca S-RNase* and *SFBB* sequences have been submitted to GenBank (accession numbers KP768248 (*M*. *fusca S28-RNase*), KP768249 (*M*. *fusca S-RNase*), and KP768250 (*M*. *fusca SFBB*)).

### Expression analyses of the *Prunus S-RNase*, *S-RNase* lineage, *SFB*, *SFB-like*, and *SLFL*-like genes

To estimate relative expression levels in *Prunus*, for the *S-RNase*, *S- RNase lineage*, *SFB*, *SFB-like*, and *SLFL*-like genes, we used *P*. *mume* cultivar landrace mRNA expression (SRA SRP014885, [45]) from fruit (GSM986570), stem (GSM986569), root (GSM986568), leaf (GSM986567), and bud (GSM986566). Furthermore, we used two other transcriptomes from *P*. *mume* cultivar Nanko for unpollinated pistils and pollen (DRR013977, and DRR002283, respectively; [63]). We applied the same methodology as in the *Malus* expression analyses, using as query sequences for the *S*-locus genes *S-RNase* scaffold 241.33 and *SFB* scaffold 241.2 for cultivar landrace, and *S1-RNase* (BAF91149.1), *S7-RNase* (BAF91155.1), *SFB1* (BAD08320.1) and *SFB7* (BAD08321.1) for cultivar Nanko. Since levels of polymorphism in coding regions are low within *P*. *mume* cultivars [64] for the remaining genes we used for both cultivars the same sequences: *PA1* scaffold 202.35.1, *S-RNase* lineage 1 scaffold 442.35, *SFB*-like lineage scaffold 57.55 and scaffold 57.57, and *SLFL*-like scaffold 101.195, and scaffold 241.9.

## Results

### Rosaceae *S-RNase* duplicate genes

We identify 21, six, five, 11, and seven *T2-RNase* genes in the draft genomes of *M*. *× domestica*, *P*. *persica*, *P*. *mume*, *F*. *vesca*, and *F*. *nipponica*, respectively (Table 1).

**Table 1:** *M*. *× domestica*, *P*. *persica*, *P*. *mume*, *F*. *vesca*, and *F*. *nipponica T2-RNase* genes, larger than 500 bp.

Gene&	Location	*T2-RNase* amino acid pattern 4	Intron number	IP
*M*. *domestica MDP0000682955*	chr1:22338267..22339935	-	1	9.06
*M*. *domestica MDP0000164105* {	chr1:1866066.. 1866836		1	8.94
*M*. *domestica MDC021344*. *1* +	chr1:11078800.. 11079914	-	2	8.06
*M*. *domestica MDC003135*. *1* +	chr4:<18896214.. 18896958	CVSIFL	1	8.8
*M*. *domestica MDP0000267606*	chr5:18789226.. 18793453	-	8	6.99
*M*. *domestica MDP0000236215*	chr5:18797610.. 18799502	-	5	7.49
*M*. *domestica MDP0000213741*	chr10:14197693.. 14200724	-	8	6.51
*M*. *domestica MDP0000251832A*	chr10:16073208.. 16073872	-	1	8.92
*M*. *domestica MDP0000210735A*	chr10:<16057129.. 16057893	-	1	7.71
*M*. *domestica MDP0000135121A- MDP0000191077A*	chr10:16077500.. 16078329	-	1	8.55
*M*. *domestica MDP0000301521A*	chr10:16048401.. 16049187	-	1	7.60
*M*. *domestica MDP0000826052*	chr13:4501130.. 4503209	CPSSNG	3	5.54
*M*. *domestica MDP0000184285* {	chr15:9375831.. 9376749	-	1	9.00
*M*. *domestica MDP0000160706*	chr15:9373449.. 9374358	-	1	9.01
*M*. *domestica MDP0000413951* {	chr15:9322264.. 9323181	-	1	8.93
*M*. *domestica MDP0000400831*	chr16:3068981.. 3073746	CPSSSG	4	4.56
*M*. *domestica MDC027512*.*1*	chr16:3068534.. 3069508	CPS(R/G)NG	3	4.68
*M*. *domestica MDP0000345854* (*S2-RNase* #)	chr17:21481499.. 21482326	-	1	9.09
*M*. *domestica MDP0000266136* (*S3-RNase* #)	unanchored:31129763.. 31131739	-	1	9.29
*M*. *domestica MDP0000250548A*	unanchored:63263516.. 63265109	-	1	7.54
*M*. *domestica MDP0000164359*	unanchored:7252151.. 7253066	-	1	8.8
*P*. *persica ppa011026m*	scaffold_1:27131291.. 27132588	CPSGSG	3	4.76
*P*. *persica ppa011014m*	scaffold_1:27134558.. 27136052	CPSSNG	3	5.01
*P*. *persica ppa011133m*	scaffold_5:8474328... 8475151	AQGKDN	1	8.85
*P*. *persica ppa018459m* (*S2-RNase*)	scaffold_6:26446964.. 26448303	-	2	9.31
*P*. *persica ppa024151m*	scaffold_8:19241555.. 19242552	-	2	9.33
*P*. *persica ppa009963m*	scaffold_8:17565332.. 17567646	RPSSCH	8	7.47
*P*. *mume scaffold 241*.*33* (*S-RNase*)	scaffold241_33.9:14388.. 18225	-	2	9.40
*P*. *mume scaffold 442*.*35*	scaffold442_35.0:1381191.. 1382394	-	2	8.98
*P*. *mume scaffold 202*.*35*.*1*	scaffold202_35.5:128144.. 128944	-	2	8.77
*P*. *mume scaffold 202*.*35*.*2* +	scaffold202_35.5:171196.. 183215	-	2	8.77
*P*. *mume scaffold 101*.*33*.*1*	scaffold101_33:595252.. 596037	-	1	9.00
*F*. *vesca 12961*	LG1 scf0513192:1522693.. 1523488	-	1	7.56
*F*. *vesca scf0513144*.*1* +	LG2 scf0513144:220982.. 212791	-	2	9.09
*F*. *vesca 04702*	LG4 scf0513158:3029880.. 3030999	CPSSNG	3	5.23
*F*. *vesca 04703a*	LG4 scf0513158b:3033059.. 3034531	CPSSSG	2	4.64
*F*. *vesca scf0513159*.*1*	LG4 scf0513159:1951083.. 1955585	VPGQRT	1	8.44
*F*. *vesca 27604*	LG5 scf0513122:220987.. 222000	CPSHTS	3	9.37
*F*. *vesca 26822*	LG5 scf0513128:351490.. 352739	-	2	7.09
*F*. *vesca 22609*	LG5 scf0513066:569.. 2628	-	1	8.95
*F*. *vesca 00227*	LG6 scf0512945:94300.. 95094	-	1	6.28
*F*. *vesca 00230*	LG6 scf0512945:109295.. 110059	-	1	6.25
*F*. *vesca scf0513063*.*1*	LG6 scf0513063:97878.. 98677	-	1	8.25
*F*. *nipponica* gi561674690-gi561985884-gi561957436	FNI_icon04160559.1:209; FNI_iscf00016107.1:1323	-	2	8.75
*F*. *nipponica* gi561805796	FNI_iscf00094180.1: 2189.. 4087	-	2	9.15
*F*. *nipponica* gi561877040	FNI_iscf00056473.1: 321.. 1113	-	1	6.21
*F*. *nipponica* gi561785734.a	FNI_iscf00105316.1: 149.. 1616	CPSSSG	2	4.58
*F*. *nipponica* gi561785734.b	FNI_iscf00105316.1:3729.. 4877	CPSSNG	3	5.24
*F*. *nipponica* gi561793890	FNI_iscf00094180.1: 2189.. 4087	-	2	7.09
*F*. *nipponica* gi561844698	FNI_iscf00073203.1:509.. 1142	-	1	6.50

^&^- it should be noted that alternative human-curated gene annotations have been used for these genes. A FASTA file with the curated coding region sequences is provided as supplementary material (S1 File).

Underscored are the amino acid positions that are not according to the *T2-RNase* lineage amino acid pattern 4 [CG]P[QLRSTIK][DGIKNPSTVY][ADEIMNPSTV][DGKNQST]), described in Vieira *et al*. [7].

IP- isoelectric point.

^#^'Golden Delicious' was chosen for genome sequencing. Accordingly, this cultivar has *S2-RNase* and *S3-RNase* [43].

^+^ stop codons are found in the sequence.

^{^gaps were introduced to avoid stop codons.

^-^ not found

The large number of *Malus T2-RNase* genes, compared with the other Rosaceae species, is consistent with a recent whole genome duplication event in Pyreae [43,65]. *M*. *× domestica T2-RNase* genes are located in chromosomes 1, 4, 5, 10, 13, 15, 16 and 17. In *P*. *persica* they are located in chromosome 1, 5, 6, and 8. *F*. *vesca T2-RNase* genes are located in chromosomes 1, 4, 5, and 6 (Table 1). Amino acid pattern 4 is absent in all *S*-lineage genes [4,7]. Therefore, the presence of this pattern in *M*. *domestica MDP0000826052*, *M*. *domestica MDP0000400831*, *P*. *persica ppa011026m*, *P*. *persica ppa011014m*, *F*. *vesca 04702*, *F*. *vesca 04703a*, *F*. *nipponica gi561785734*.*a*, and *F*. *nipponica gi561785734*.*b* genes (Table 1), exclude them as *S*-lineage genes. Furthermore, *S-RNases* present at maximum two introns [5,52]. Thus, the presence of more than two introns in *M*. *domestica MDP0000267606*, *M*. *domestica MDP0000236215*, *M*. *domestica MDP0000213741*, *M*. *domestica MDC027512*.*1*, *P*. *persica ppa009963m*, and *F*. *vesca 27604* genes, strongly suggests that these genes are also not *S*-lineage genes.

**Fig 1:**
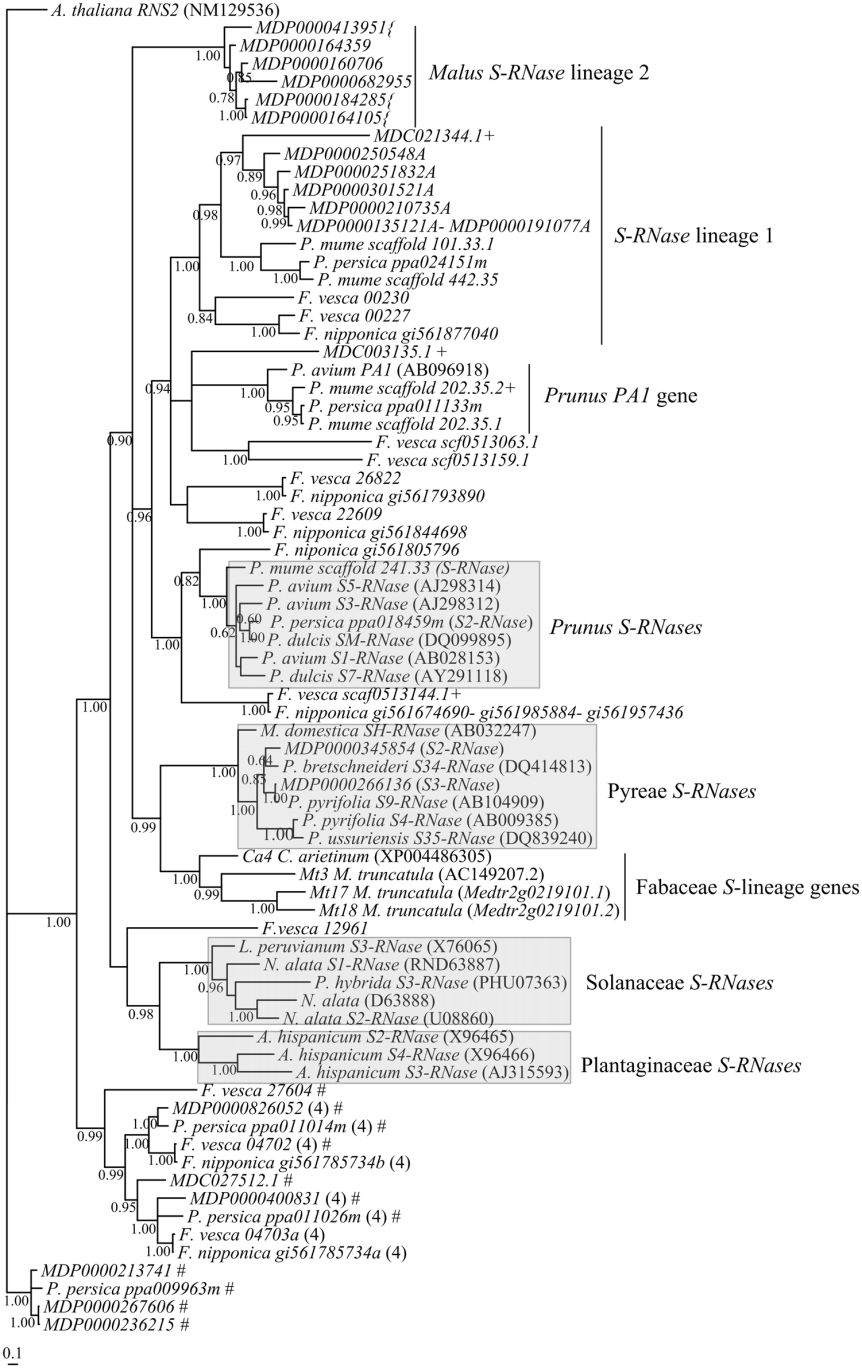
Bayesian phylogenetic tree of Rosaceae *T2*-*RNase* lineage, using T-coffee alignment method. The tree shows the relationship of the *M*. *domestica* (*M*. *domestica MDP/MDC*), *P*. *persica* (*P*. *persica ppa/ppb*), *P*. *mume* (*P*. *mume scaffold*), *F*. *vesca*, and *F*. *nipponica T2*-*RNase* lineage genes. The tree was rooted with *T2-RNase A*. *thaliana RNS2* (NM129536). Numbers below the branches represent posterior credibility values above 60. In grey are the reference sequences (*S-RNases* from *Prunus*, Pyreae, Solanaceae, and Plantaginaceae, and Fabaceae *S*-lineage genes). # indicate sequences with more than two introns; (4) the sequences that show in the putative protein sequence amino acid pattern 4, that is absent in all *S*-lineage genes [4,7]; the + indicate sequences that present stop codons; the {indicate sequences where gaps were introduced to avoid stop codons.

The phylogenetic relationship of the Rosaceae *T2-RNase* sequences here identified, together with previously described *Malus*, *Prunus*, Solanaceae, and Plantaginaceae *S-RNases*, and Fabaceae (*Medicago truncatula*, and *Cicer arietinum*) *S*-lineage genes (*S-RNase* like genes of self-compatible species, that are surrounded by F-box *Malus SFBB*/ *Prunus SLFL* like sequences, and are expressed in tissues other than pistil; unpublished results) is presented in Fig 1 (see also S1 Fig). The phylogeny supports the inferences that sequences presenting amino acid pattern 4, and/or more than two introns, are not *S*-lineage genes, since they do not cluster with *Malus*, *Prunus*, Solanaceae, and Plantaginaceae *S-RNases*.

**Fig 2:**
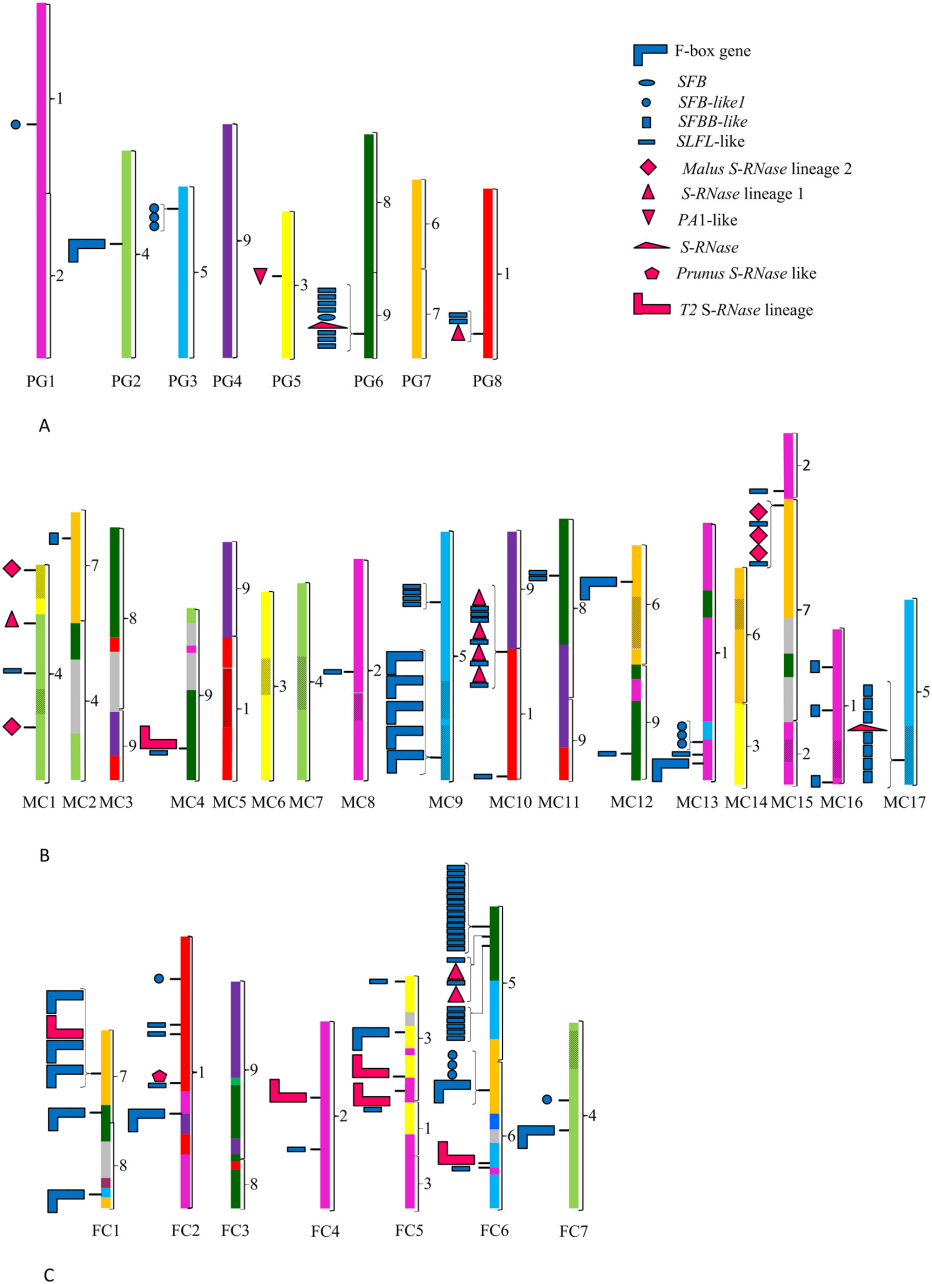
Chromosomal localization of the *S-RNase*, *SFB*, *SFBB*, and *SLFL* lineage genes. *P*. *persica* (A), *M*. *domestica* (B), and *F*. *vesca* (C) *S-RNase* lineage genes are marked in pink, *SFB*, *SFBB*, and *SLFL* lineage genes are marked in blue. Different shapes represent the different *S-RNase* and F-box *SFB-*, *SFBB-*, and *SLFL-* lineage genes. To represent two or more sequential genes, a bracket at the left of the chromosome is used. Each *Prunus* chromosome is marked in a different colour: PG1- pink, PG2 light green, PG3 light blue, PG4- purple, PG5- yellow, PG6-green, PG7- orange, and PG8-red. These colours are then used to assign the synteny regions for the *M*. *domestica* and *F*. *vesca* chromosomes, according to Fig 1in Jung *et al*., [65]. Regions with unknown synteny but between regions that show synteny with the same chromosome are marked in stripes, and regions with unknown synteny between syntenic regions from different chromosomes are marked in grey. Brackets on the right of each chromosome represent the nine ancestral synteny regions (1 to 9) according to Fig 4 in Illa et al. [72].

The *Malus S-RNase* lineage genes defined three groups: Pyreae *S-RNases*, *S*-*RNase* lineage 1, and *Malus S*-*RNase* lineage 2 (Fig 1; S1 Fig). The two *S-RNases* present in the sequenced *M*. *× domestica* cultivar, according to blastn results, are *S2-RNase*, and *S3-RNase*. Although the sequenced scaffold containing the *Malus S3-RNase* is not anchored, the *S2-RNase* is located on *Malus* chromosome (MC) 17 (Table 1; Fig 2), in agreement with the linkage-based results [66]. *Malus S-RNase*s cluster with Fabaceae *S*-lineage genes, and not with *Prunus S-RNases*. Thus, we can conclude that *Malus* and *Prunus S-RNase* genes represent two different gene lineages.

Four out of five *Malus S*-*RNase* lineage 1 putative functional genes are located on MC10 (Table 1; Figs 1 and 2). These four genes have been wrongly annotated due to the incorporation of exons from a different gene into a single one (http://www.rosaceae.org/). Indeed, our transcriptional data (below) supports our inference that they represent two genes, namely one *S-RNase* lineage gene and one F-box gene. Here we use the gene name and the suffix-A for the *S-RNase* duplicate and-B for the F-box gene. *Malus S-RNase* lineage 2 genes are located at MC1 and MC15 (Table 1; Fig 2). These regions represent different linkage groups in the putative Rosaceae ancestral genome (Fig 2).

*Prunus S-RNase* lineage genes also defined three groups: the *Prunus S-RNase* gene, the *S-RNase* lineage 1 (that cluster with *Malus S-RNase* lineage 1 genes), and a *Prunus* specific group that includes the *P*. *avium PA1* ([67]; Fig 1; S1 Fig). In *P*. *mume*, a self-incompatible species, only one of the *S-RNase* alleles was obtained. By performing blastn, it is clear that this *S-RNase* sequence has not been previously identified. It should be noted that, chromosomal location is only available for *P*. *persica*, a self-compatible (SC) species. SC in *Prunus* has been achieved by non-sense mutations mainly at the *SFB* gene, but also at the *S-RNase* gene [24], mutations producing low *S-RNase* transcription levels [68], and mutations at loci unlinked to the *S*-locus [69,70]. In *P*. *persica* we identify the *S2-RNase* that does not have in frame stop codons. According to the location of the *P*. *persica S2-RNase* allele, the *S*-locus region is at scaffold 6, as reported by Dirlewanger et al. [71]. This scaffold shows synteny to *Malus* MC2/MC15, MC3/MC11, and MC4/MC12, but not to MC17 [65,72]. Indeed, when the 194 Kb region flanking the *Prunus S2-RNase* is used as query against the *M*. *x domestica* genome two hits are observed with *M*. *x domestica* chromosome 4 and chromosome 12 (S2 Fig). It should be noted that, in *Malus*, because of a whole genome duplication, large syntenic regions are found between two or more chromosomes (Fig 2, [65]). Thus, the *S*-locus regions in *Prunus* and *Malus* represent different linkage groups in the putative Rosaceae ancestral genome (Fig 2). *P*. *avium PA1* [67] in two of the alignment methods clusters with *S*-*RNase* lineage 1 genes. Thus, it may represent a duplication of this gene lineage and not a duplication of the *Prunus S-RNase* gene [67]. Like in *Malus*, *P*. *persica ppa024151m S-RNase* lineage 1 gene has in its vicinity an F-box gene (*P*. *persica ppa024694m*; S3 Table). This gene is located at *Prunus* scaffold PG8, a linkage group syntenic with *Malus* MC10 (and also MC5, and MC3/MC11) [65,72] where *Malus S*-*RNase* lineage 1 genes are located. These regions represent the putative Rosaceae ancestral linkage group1 (Fig 2).

There are *Fragaria S-RNase* lineage genes that cluster with *Prunus S-RNases* (the *F*. *nipponica gi561805796*, *F*. *nipponica gi561674690- gi561985884- gi561957436*, and the pseudogene *F*. *vesca scf0513144*.*1*), but none of the *Fragaria* genes cluster with the *Malus S-RNase* gene (Fig 1; S1 Fig). It should be noted that *F*. *nipponica* is a SI species [73], that shows three bands in zymograms [49], and *F*. *vesca* is a SC species that lacks stylar *S-RNase* activity [49]. In *Fragaria* two unlinked *RNase* loci, the *S*-locus (FC1) and *T*-locus (FC6) have been described, although a single active allele at either of the two loci is sufficient to confer self-incompatibility [49]. Based on the ribonuclease zymograms, *Fragaria T2-RNases* can show high IP, as those at the *S*-locus (located at FC1; [49]), characteristic of *S-RNases* [3], but they can also be at the base of the cathodal region, as those at the *T*-locus (located at FC6;[49]), thus presenting a neutral IP. The *F*. *nipponica gi561805796*, and *F*. *nipponica gi561674690-gi561985884-gi561957436* sequences, that cluster with the *Prunus S-RNase*, encode proteins with an isoelectric point above 9 (Table 1) and thus, in principle, can only represent alleles of the *Fragaria S*-locus *S-RNase* gene. Moreover, these sequences show two introns at the same positions as the *Prunus S-RNase* gene [52]. Nevertheless, the presence of *F*. *vesca scf0513144*.*1* sequence that shows low levels of divergence with *F*. *nipponica gi561674690- gi561985884- gi561957436* (*K*_*s*_ in coding regions is 0.039, after Jukes Cantor correction) suggests that this *F*. *nipponica* sequence is not an *S-RNase* allele, but a *S*-lineage gene. Indeed, using blastn and *Fragari*a whole genome shotgun contigs (wgs) Database, this gene is highly conserved in *Fragaria* (nucleotide identity higher than 98% in *F*. *ananassa* (dbj|BATT01112757.1|), *F*. *nubicola* (dbj|BATW01064019.1|), and *F*. *orientalis* (dbj|BATX01305571.1)). Moreover, *F*. *vesca scf0513144*.*1* is located at linkage group FC2 (Fig 2), and the *Fragaria S*-locus has been assigned to FC1[74]. Therefore, only the *F*. *nipponica gi561805796* gene may represent one of the *Fragaria S*-locus pistil genes, but unfortunately genomic location of this gene is not available in *F*. *nipponica* since the contig where it is located is short (4292 bp). Since *F*. *vesca* is a self-compatible species and thus the *S*-locus region may be deleted or non-functional, and the *F*. *nipponica gi561805796* sequence clusters with *Prunus S-RNases*, we also performed a blastn search using the 194 Kb *Prunus S2-RNase* flanking region as query against the *F*. *vesca* genome. Two regions were identified showing synteny, one at FC1, and another at FC6, the two regions identified by Bošković *et al*. [49], as the regions harbouring the *S*- and *T*- locus, respectively (S3 Fig). The syntenic region located at FC1 is approximately at the middle of chromosome 1 and the region located at FC6 is approximately located at one end of chromosome 6 near the *Pgl1* gene, which is broadly compatible with the locations of the *S*- and *T*- loci based on the recombination map shown by Bošković *et al*. [49]. Therefore, levels of diversity for the *F*. *nipponica gi561805796* gene together with segregation analyses and genomic localization are needed to determine if this is indeed the *Fragaria S*-locus S-*RNase* gene.

To identify other *Fragaria T2-RNase* gene candidates we also looked at levels of nucleotide similarity among *Fragaria* species for the other *T2-RNase*-lineage genes at FC1 and FC6. Since a single active allele at either of the two loci is sufficient to confer self-incompatibility [49], both loci must be under positive selection and thus, levels of diversity at each of the *T2-RNase* genes is expected to be large. In FC1, there is only one *S-RNase*-lineage gene (Fig 2), namely *F*. *vesca 12961* (Table 1). This gene codes for a putative *T2-RNase* with a neutral IP (Table 1), and basic ribonucleases have been assigned to FC1. The phylogenetic relationship of this sequence depends on the alignment method used (Fig 1, and S1 Fig). Nevertheless, this gene is highly conserved (nucleotide identity higher than 98%) in *F*. *ananassa* (dbj|BATT01682710.1|), *F*. *nubicola* (dbj|BATW01027551.1|), and *F*. *orientalis* (dbj|BATX01257665.1|). Moreover, the *F*. *vesca 12961* gene is not surrounded by *F*-box genes showing similarity to either *Malus* or *Prunus S*-pollen genes (Fig 2), and thus it is unlikely to be the *Fragaria S-RNase S*-locus gene. Located in FC6 there are three genes, namely *F*. *vesca 00227*, *F*. *vesca 00230*, and *F*. *vesca scf0513063*.*1* (Table 1), that could represent *T2-RNases* of the *T*-locus. *F*. *vesca 00227* gene is highly conserved (nucleotide identity higher than 98%) in *F*. *ananassa* (dbj|BATT01017701.1|), *F*. *nubicola* (dbj|BATW01044818.1|), and *F*. *orientalis* (dbj|BATX01013415.1|). For *F*. *vesca 00230* gene, high nucleotide homology (more than 97%) is observed in *F*. *iinumae* (dbj|BATU01072984.1|), *F*. *ananassa* (dbj|BATT01231756.1|), *F*. *nubicola* (dbj|BATW01024410.1|), and *F*. *orientalis* (dbj|BATX01105279.1|). Therefore, it is unlikely that these genes represent the *Fragaria T2-RNase T*-locus gene. For *F*. *vesca scf0513063*.*1* gene, levels of similarity with other *Fragaria* species is below 96% (*F*. *iinumae* (dbj|BATU01063406.1|), and *F*. *ananassa* (dbj|BATT01457356.1|)). When the 5´region of *F*. *iinumae* dbj|BATU01063406.1contig (427 bp long) is blasted against the *F*. *vesca* genome, only one hit is obtained with *F*. *vesca scf0513063*, with 94% homology. Similar result is observed with 3' region of *F*. *ananassa* dbj|BATT01457356.1| contig. Therefore, these *T2-RNases* seem to be orthologous. The *F*. *vesca scf0513063*.*1* gene can thus, represent the *T2-RNase* at the *T*-locus. Indeed, this gene has in its vicinity a *F*-box gene (Fig 2). Nevertheless, this region is not located on the FC6 region identified by Bošković *et al*. [49] as being the *T*-locus region. Indeed, the *T*-locus region is near the *Pgl1* gene and the *F*. *vesca scf0513063*.*1* gene is located on the other side of the chromosome. It should also be noted that the *F*. *vesca scf0513063*.*1* gene is not located in the *F*. *vesca* region that shows synteny with the *Prunus S*-locus flanking region and that is near the *Pgl1* gene (see above). None of the sequences here identified as the putative *Fragaria S*- an *T*- locus share homology with the S-RNase peptide sequences identified by Bošković *et al*. [49]. Indeed, these peptide sequences show similarity with the protein encoded by *F*. *vesca 17424* (LG2:9,443,884..12,126,193) gene that belongs to the Glo_EDI_BRP_like superfamily.

Since for *F*. *nipponica* contigs, chromosomal location is not available, we address also if *F*. *nipponica gi561793890*, *F*. *nipponica gi561844698*, and *F*. *nipponica gi561877040 T2-RNase* sequences could represent the *T2-RNases* of the *T*-locus. For both *F*. *nipponica gi561793890* and *F*. *nipponica gi561844698* low levels of divergence were observed with *F*. *vesca 26822* and *F*. vesca *22609*, respectively (Fig 1). These *F*. *vesca* genes are located at LG 5 (Table 1). Furthermore, *F*. *nipponica gi561793890* is highly conserved (nucleotide identity higher than 97%) in *F*. *nubicola* (dbj|BATW01019403.1|), *F*. *orientalis* (dbj|BATX01097770.1|), and *F*. *ananassa* (dbj|BATS01008420.1|). High conservation is also observed between *F*. *nipponica gi561844698 T2-RNase* and sequences of *F*. *orientalis* (dbj|BATX01070344.1|), and *F*. *ananassa* (dbj|BATT01173691.1|). Thus, it is unlikely that these genes are the *Fragaria S-RNase T*-locus gene. The *F*. *nipponica gi561877040* sequence is 1394 bp long and the *T2-RNase* gene is 793 bp long. High nucleotide conservation (99%) is observed only with *F*. *ananassa* (dbj|BATT01303160.1|) at the entire region. Such high homology is not expected between different *S-RNase* alleles, but this *F*. *ananassa* individual could share the same *S-RNase* allele with that of *F*. *nipponica*. When the *F*. *nipponica gi561877040 T2-RNase* region is used as query against the *F*. *vesca* genome using blast, 90% homology is observed with *F*. *vesca scf0512945*. This region contains the *F*. *vesca 00227* gene, that in the phylogenetic analyses clusters with *F*. *nipponica gi561877040 T2-RNase* (Fig 1). When the same approach is used but the flanking regions of *F*. *nipponica gi561877040 T2-RNase* are used as query, high homology (96%) is observed with *F*. *vesca scf0513158_5*. A higher homology (99%) is observed with this *F*. *vesca* contig, using as query the larger *F*. *ananassa* contig (5740 bp long). *F*. *vesca* scf0513158_5 is located at LG5. Therefore, there is no evidence for this sequence being the *T*-locus *RNase* gene in *Fragaria*.

In summary, given the close relationship of *F*. *nipponica gi561805796* gene with *Prunus S-RNase* gene, and the presence of Fabaceae *S*-lineage genes that cluster with *Malus S-RNases*, we can conclude that *Malus* and *Prunus S-RNase* genes represent two different gene lineages, and that *Fragaria* is most similar to *Prunus*. Moreover, the regions where *Malus* and *Prunus S*-locus are located are not orthologous.

### *Malus*, *Prunus* and *Fragaria SFB*, *SLFL* and *SFBB*—like genes

**Fig 3:**
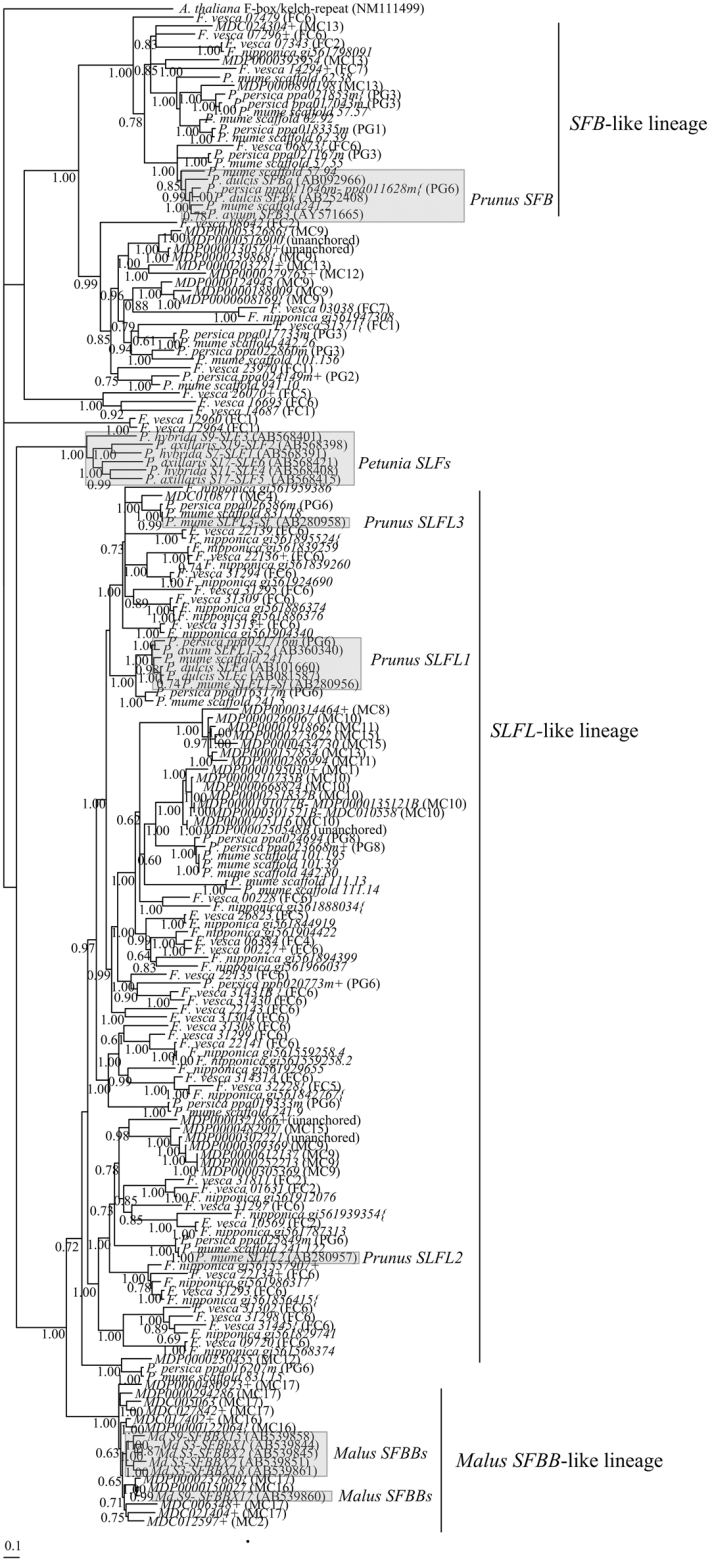
Bayesian phylogenetic tree of Rosaceae *SFB-* and *SFBB-*like genes, using T-coffee alignment method. The tree shows the relationship of the *M*. *× domestica* (*M*. *domestica MDP/MDC*), *P*. *persica* (*P*. *persica ppa/ppb*), *P*. *mume* (*P*. *mume scaffold*), *F*. *vesca*, and *F*. *nipponica* F-box *SFBB-* and *SFB-* like genes. In grey are the reference sequences (*Prunus SFB*, *Prunus SLFLs*, Pyreae *SFBB*s, and *Petunia SLF* genes). The tree was rooted with *A*. *thaliana* F-box/kelch-repeat gene (NM111499). Numbers below the branches represent posterior credibility values above 60. The + indicate sequences that present stop codons; the {indicates sequences where gaps were introduced to avoid stop codons. In brackets are indicated the chromosomal location of *M*. *domestica*, *P*. *persica* and *F*. *vesca* genes (S2, S3, and S5 Tables).

A total of 84, 45, 56, 85, and 55 *SFB* and *SLFL*—like sequences were derived from the draft genomes of *M*. *× domestica*, *P*. *persica*, *P*. *mume*, *F*. *vesca* and *F*. *nipponica*, respectively (S2, S3, S4, S5, and S6) Tables. Of these, only 49 *M*. *× domestica*, 17 *P*. *persica*, 22 *P*. *mume*, 44 *F*. *vesca* and 27 *F*. *nipponica* genes, show a close relationship with *Prunus SFB*, *Malus SFBBs*, and *Petunia SLFs* genes (known to be involved in GSI specificity determination; see reviews by Tao and Iezzoni [75], and De Franceschi et al. [76]), and *Prunus SLFL* genes (not involved in GSI specificity determination, but that are located in the region surrounding the *S*-locus; S4, S5, and S6 Figs). Not all of them seem to be functional, since they contain in-frame stop codons or insertion/deletions that disrupt the ORF (S2, S3, S4, S5, and S6 Tables). The phylogenetic relationship of the selected *SFB-*, *SLFL-* and *SFBB*- like sequences from the five genomes (Fig 3; see also S7 Fig), defined three main Rosaceae gene lineages, namely: 1) *Malus SFBB*-like gene lineage; 2) *SFB*-like gene lineage, and 3) a large *SLFL*-like gene lineage.

Eleven genes cluster with the *Malus SFBB* reference genes (Fig 3). Seven *SFBB*-like genes are from MC17 (the location of the *Malus S*-locus; Fig 2), but only two (*MDC005063* and *MDP0000294286*) seem to be functional. The other genes are likely non-functional since alignment gaps had to be included to put the sequence back into the right frame, or have in frame stop codons (S2 Table). The exact number of *Malus SFBBs* is unknown, but more than 10 genes have been described at the *S*-locus [22,77]. Therefore, *S*-pollen genes are poorly represented in the *Malu*s genome sequence. It should be noted that *MDP0000150027* gene located at MC16 codes for a protein that is identical to SFBBX17 (BAJ11965), that shows linkage with the *S9-RNase* [22], and thus, it must be erroneously located. The other three *SFBB*-like genes located at MC2 and MC16 (Fig 2) are likely non-functional since alignment gaps had to be included to put the sequence back into the right frame, or have in frame stop codons (S2 Table). The presence of *SFBB*-like sequences in regions other than the *S*-locus implies that phylogenetic analyses alone may lead to the incorrect assignment of *SFBB*-like genes to the *S*-locus. Therefore, linkage analyses with the *S-RNase* gene are needed for the identification of *SFBB* genes. *Malus SFBB* genes are closely related to *Prunus* and *Fragaria SLFL*-like genes (Fig 3), as described before [19,28,34–36]. *Malus SFBB* genes are, however, a distinct lineage since there are other *Malus SLFL* genes that are more closely related to *Prunus* and *Fragaria SLFL* genes than *Malus SFBB* genes. Furthermore, *Malus SLFL*-like lineage genes are not present in the *Malus S*-locus region (MC17; Fig 2).

*Malus SLFL*-like genes are a very heterogeneous group of genes (Fig 3) and they are located in nine chromossomes (Fig 2; S2 Table). Nevertheless, the two *Malus SLFL*-like genes, *MDC010871* located at MC4, and *MDP0000250455* located at MC12 (Figs 2 and 3) that are orthologs of *Prunus SLFL* genes located in the vicinity of the *S*-locus region (*SLFL3* and *P*. *persica ppa016207m*) are located in syntenic regions. This suggests the presence of *SLFL*-like genes similar to those of the *Prunus S*-locus in the ancestral Rosaceae group 9.

Here we report *Malus SFB*-like sequences for the first time. *MDP0000890198*, *MDC024304*, and *MDP0000393954* genes, located at MC13, cluster with *Prunus* and *Fragaria SFB*-like genes. These *Malus SFB*-like genes represent, however ancient gene duplications that occurred before the separation of *Fragaria* and the *Malus*/*Prunus* lineage. They could represent the *SFB*-like genes of the ancestral Rosaceae group 1 (Fig 2; [65,72]).

In the *P*. *persica S*-locus region there is a mutated *S2*-*SFB* gene (*P*. *persica ppa011628m*; a 5 bp sequence is inserted in the *S2-SFB* gene) that has been reported to cause SC in this species [24]. The *P*. *mume scaffold 57*.*94* and *P*. *mume scaffold241*.*2* are the two *SFB* alleles of the cultivar sequenced (Fig 3), and have not been described before. *Prunus SFB* gene is clearly a distinct lineage of the *Malus SFBB* genes (Fig 3). Although there is a *SFB*-like gene closely related to *Prunus SFB* gene (represented by *P*. *persica ppa021167m*, and *P*. *mume scaffold 57*.*55* sequences; Fig 3), it is easily distinguished from the *SFB* gene since it presents low sequence divergence. Furthermore, this gene in *P*. *persica* is located in PG3 (S3 Table). In the vicinity of the *Prunus S*-locus, there are seven *SLFL* genes: three previously reported—*SLFL1* (*P*. *persica ppa021716m*), *SLFL2* (*P*. *persica ppa025849m*), *and SLFL3* (*P*. *persica ppa026586m*; [12,13]), three unreported *SLFL*s genes-*P*. *persica ppa019333m*, *P*. *persica ppa016317m*, and *P*. *persica ppa016207*, and one unreported putative *SLFL* pseudogene (*ppb020773m+*).

In *Fragaria*, the number of *SLFL*-like genes is larger than in *Prunus* and *Malus* (Fig 3). About 70% of those are located at FC6 (Fig 2) and are the result of two tandem duplications (Fig 2; [65,72]). Only *F*. *vesca 06873* clusters with *Prunus SFB*, and *SFB*-like *P*. *persica ppa021167m*, and *P*. *mume scaffold 57*.*55* genes (Fig 3). Although *F*. *vesca 06873* is located at FC6 (that is syntenic to PG6, where the *Prunus S*-locus is located; Fig 2, and S3 Fig), it shows 98% nucleotide identity with sequences from other *Fragari*a species (*F*. *nubicola* BATW01053320.1; *F*. *iinumae* BATU01056017.1). Thus, *F*. *vesca 06873* pseudogene represents a *SFB*-like gene, and not a non functional *S*-pollen gene. None of the *F*. *vesca* F-box *SFBB*- *SFB*- *SLFL*- like genes located in FC1 region cluster with *Prunus* or *Malus S*-pollen genes, as observed for the *T2-RNases*.

In conclusion, the evidence here presented implies that *Prunus SFB* gene is from a distinct phylogenetic clade of the *Malus SFBB* genes. Furthermore *Malus SFBB* genes are a sister clade of *Malus* and *Prunus SLFL* genes. Although no putative *Fragaria S*-pollen has been identified, the presence of *SLFL*-like genes orthologous of the *Prunus SLFL* genes located in the vicinity of the *Prunus S*-locus, at FC6, suggests a similar organization to that of *Prunus S*-locus for the *Fragaria T*-locus. This region is the same that is identified when using the 194 Kb *Prunus* region flanking the *S2-RNase* (see above). Furthermore, we found no evidence for *Malus SFBB* lineage genes in *Fragaria*.

### *Malus* and *Prunus* expression analyses of the *S- RNase*, *S- RNase* lineage, *SFB*, *SFB-like*, *SFBB*, and *SLFL*-like genes

In order to understand how difficult it is to evolve the restricted expression pattern shown by *S*-pistil and *S*-pollen genes, inferences must be made on the expression of the ancestral *S-RNase* lineage and *S*-pollen lineage genes. We addressed the expression of these lineage genes using RNA-seq expression data. In *Malus* we analysed 17 tissues derived from developmental transitions of the wild apple *M*. *fusca* (S1 Table), and in *Prunus*, seven tissues from two *P*. *mume* cultivars (Material and Methods).

**Fig 4:**
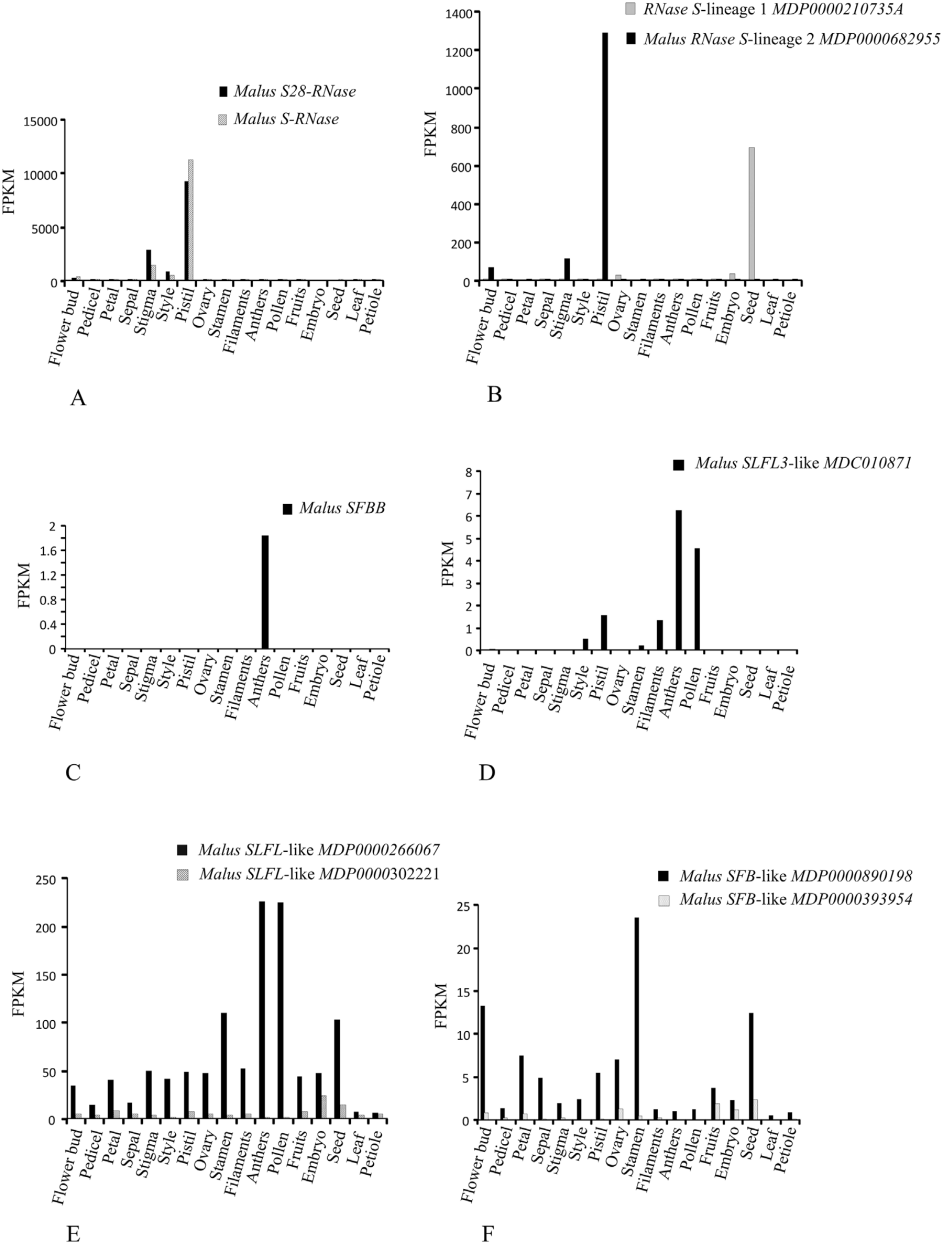
*M*.*fusca* expression levels (FPKM) for *S-RNase*, *SFB*, *SFBB*, and *SLFL* lineage genes in 17 tissues. (A) *S- RNase*, (B) *S- RNase* lineages, (C) *SFBB*, (D) *SLFL3*-like, (E) *SLFL*-like, and (F) *SFB*-like genes.

**Fig 5:**
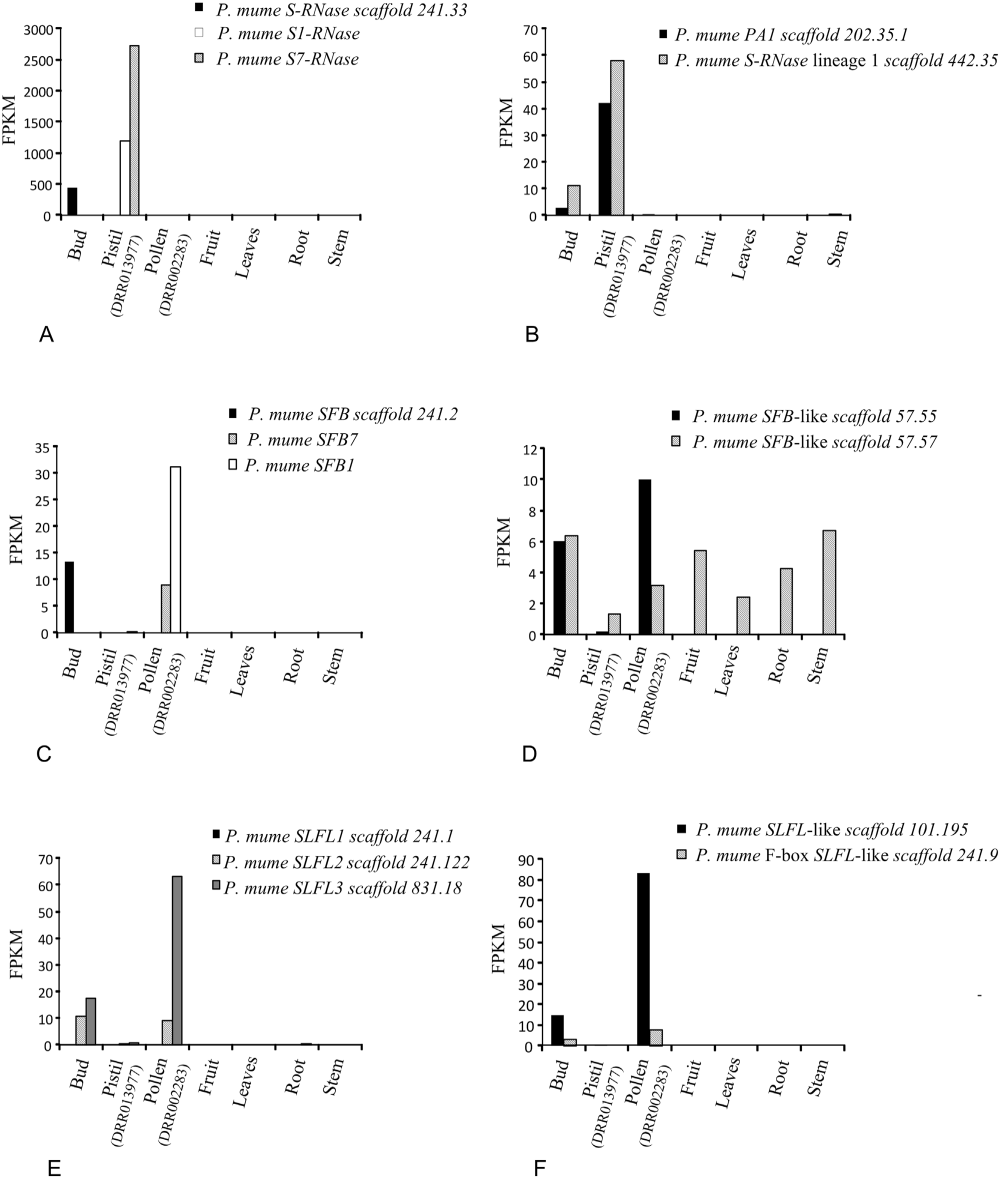
*P*. *mume* expression levels (FPKM) for *S-RNase*, *SFB*, *SFBB*, and *SLFL* lineage genes in 7 tissues. (A) *S- RNase*, (B) *S- RNase* lineages, (C) *SFB*, (D) *SFB*-like, (E) *SLFL1-*, *SLFL2- SLFL3*-like, and (F) other F box *SLFL*-like (F) gene lineages.

*Malus S-RNase* gene is most highly represented in whole pistils one week prior to anthesis, in stigmas and styles of flowers at anthesis (Fig 4A), as described before [78], but they also show low expression in entire flower buds. *Malus S*-*RNase* lineage 2 gene, shows a similar pattern of expression to the *S-RNase* gene (maximum expression in pistils one week prior to anthesis, much less in stigma of flowers at anthesis, and entire flowers buds; Fig 4B), but levels of expression are about seven times lower than for the *S-RNase*. In contrast, *S*-*RNase* lineage 1 genes show maximum expression in seeds, and moderate expression in embryos and ovary (Fig 4B). *Prunus S-RNases*, *S-RNase* lineage 1, and *PA1*genes are also expressed in the pistil and buds (Fig 5A and 5B). Therefore, the ancestral *S-RNase* gene is inferred to show expression mainly in pistils but also, at lower amounts, in stigmas, styles of flowers at anthesis and flowers buds.

Expression of *SFBB* genes is reported to be pollen-specific [21,22,26,27,76]. Accordingly, we found expression in anthers at anthesis (Fig 4C). It should be noted that *SFBB* expression is 5000 times lower than that of the *S-RNase* gene. *SLFL*-like genes are a very large group of genes, and thus, they show different patterns of expression. They can show maximum expression in anthers and pollen such as *MDC010871* (Fig 4D), and *MDP0000266067* (Fig 4E), but can be also expressed in a few other tissues (pistil, style stamen and filaments) such as *MDC010871* (Fig 4D), or they can show moderate expression in most of the tissues analysed, such as the *MDP0000266067* gene (Fig 4E). They can even show no expression in anthers and pollen such as *MDP0000302221* (Fig 4E), but show low expression in most of the tissues analysed. *Prunus SLFL* genes are expressed in pollen (Fig 5E), as described before [12,13], in flowering buds, but also, very little, in the pistil. Expression in pollen and buds are also observed for other *Prunus SLFL*-like genes (Fig 5F). Thus, from this data it is not possible to infer the expression of the ancestral *SFBB* gene.

Although *Prunus SFB* gene has been reported as highly expressed in pollen and anthers [12], it shows 70 times less expression than the *Prunus S-RNase* gene (Fig 5C). This gene is also expressed in buds. Similar expression pattern is observed for the closely related *SFB*-like *P*. *mume scaffold 57*.*55*. More divergent *SFB*-like genes have a different expression pattern (see for instance *P*. *mume scaffold 241*.*9*, (Fig 5D) and *Malus MDP0000890198* (Fig 4F) genes). We can infer that the ancestral *Prunus SFB* gene would show a similar expression to that of *Prunus SFB*.

## Discussion

*S-RNase* based GSI, according to the *S-RNase* gene has evolved about 120 MY ago, and thus is expected to be shared among distantly related species. Our results show that, in *Malus* and *Prunus* the *S-RNase* and the *S*-pollen genes have evolved from paralogous genes. The *Malus S-RNase* and *SFBB* gene lineages are not present in *Prunus*, and *Fragaria*. The presence of the *F*. *nipponica gi561805796 Prunus S-RNase* lineage gene suggests that *Fragaria* and *Prunus* share a common ancestral *S*-locus region. The location of this gene is unknown, but given its basic IP, it could represent the *Fragaria S*-locus. The location of this gene must be however confirmed. The presence of *SLFL-* like genes in *Fragaria* FC6 that cluster with *Prunus SLFL*- like genes surrounding the *S*-locus region, as well as the conservation between the *Prunus S*-locus flanking regions and this FC6 region, further suggests that the *T*-locus could show a similar organization to that of *Prunus S*-locus. This region is located near the *Pgl1* gene and thus corresponds to the *T*-locus identified by Bošković *et al*. [49]. The *F*. *vesca scf0513063*.*1* gene identified as a *T2-RNase T*-locus candidate gene, is, however, not located in the region identified by Bošković *et al*. [49] as being the *T*-locus, and thus should be treated with caution.

The hypothesis that *Fragaria* and *Prunus* share a common ancestral self-incompatibility locus organization is unexpected because of the similarities between the *Petunia* and *Malus S-RNase* based GSI mechanism of pollen recognition by multiple *S*-pollen genes [19–23,26,28,29,76]. Thus, in *Fragaria* that is an out group to *Prunus* and *Malus*, the *S*-locus genes were expected to belong to the *Malus S*-lineage. It could be that in the ancestral Rosaceae, there were three loci determining GSI, the *S* and *T Fragaria* loci and the *Malus S*-locus. Under this scenario, different GSI loci were retained and lost in different Rosaceae SI species.

One alternative hypothesis is that the *Malus S*-locus region could have evolved *de novo* from a partial duplication of the ancestral *Fragaria*/*Prunus*-like *S*-locus region. Under this hypothesis the *S*-*RNase* duplicate gene is the *Malus* pistil component and duplicates of the ancestral *SLFL—*like genes (*Malus SFBB* genes) are now the *S*-pollen genes. It is likely that the ancestral *SLFL*-like genes were expressed in pollen, but also in other tissues and thus, they could have evolved an expression restricted to pollen and anthers. Further genomic data from other SI Rosaceae species, as well as a better assembly for *Fragaria* SI species, is needed to distinguish the two hypotheses.

The molecular characterization of *S-RNase* based GSI often starts with a search for *S*-*RNase* lineage genes, since they are just a few. Moreover, *S-RNase* lineage genes can be easily distinguished from other *T2-RNases* by looking at the sequences of the proteins they encode. Indeed, Vieira et al. [7] have shown that amino acid patterns 1 and 2 are exclusively found in proteins encoded by *S*-lineage genes, while pattern 4 is not found in any protein encoded by *S*-lineage genes. Moreover, in contrast to other *T2-RNases*, *S-RNase* lineage sequences have just one or two introns [5] and encode proteins with a basic isoelectric point [3]. Nevertheless, phylogenetic analyses of the *S-RNase* lineage genes alone is not enough to identify the *S-RNase* gene. For instance, in *Coffea* (Rubiaceae) there are at least three distinct *S-RNase* lineage genes [4]. The secondary evolution of GSI from paralogous regions further complicates the identification of the *S-RNase* gene. Thus, identifying the GSI biochemical components in non characterised eudicot species may be more difficult than anticipated. The expression pattern is not enough either, since, as we here show, there are *S-RNase* duplicates with an expression pattern identical to that of the *S-RNase* gene. Therefore, besides phylogenetic and expression analyses, evidence for high polymorphism levels, positively selected amino acid sites, as well as segregation analyses in controlled crosses are needed to identify the *S-RNase*. It should be noted that in the Pyreae *S*-locus region there are likely also genes that perform functions unrelated to self-incompatibility [21,27]. Such genes might contribute to other phenotypes of agronomical interest. In Rosaceae species where the *S*-locus is large, as in Pyreae, variants at these genes will co-segregate with GSI specificities [79]. It is thus, important to determine whether the *S*-locus structure of most Rosaceae species is similar to that present in Pyreae.

In conclusion, *S-RNase* based GSI may evolve multiple times from *S*-locus paralogous regions, as it happens in Rosaceae. In Brassicaceae, a duplication event of the *S*-locus region and recruitment of the paralogous genes of the ancestral SSI *Arabidopsis* and *Brassica S*-locus genes that determine SI has been described also in *Leavenworthia* [39]. Thus, multiple independent recruitment of SI genes from the same gene families may be an unexpected but common evolutionary process in plant SI systems.

## Supporting Information

S1 Fig Bayesian phylogenetic tree of Rosaceae *T2*-*RNase* lineage using ClustalW2 (A), and Muscle (B) alignment algorithm. The trees show the relationship of the *M*. *x domestica* (*MDP/MDC*), *P*. *persica* (*P*. *persica ppa/ppb*), *P*. *mume* (*P*. *mume scaffold*), *F*. *vesca*, and *F*. *nipponica T2-RNase* lineage genes. Legend as in Fig 1.
(TIF)

S2 Fig Dot matrix view showing the conservation of the *Prunus S*-locus and flanking regions in *M*. *x domestica*. The *Prunus* region is that in between the *ppa019333m* (*SLFL-like*) and *pp016207m* (*SLFL-like*) (see S3 Table) while the *Malus* region is that in between position 19264736 to19566015 (MC4; panel A) and position 27854860 to 28144804 (MC12; panel B).
(TIF)

S3 Fig Dot matrix view showing the conservation of the *Prunus S*-locus and flanking regions in *F*. *vesca*. The *Prunus* region is that in between the *ppa019333m* (*SLFL-like*) and *pp016207m* (*SLFL-like*) (see S3 Table) while the *Fragaria* region is that in between position 13347728 to13464174 (FC1; panel A) and position 2001274 to 2067898 (FC6; panel B).
(TIF)

S4 Fig Bayesian phylogenetic tree of *M*. *x domestica* (*MDP/MDC*) *SFBB-* and *SFB-* like genes. The tree shows the relationship of these genes with *Prunus SFB*, *Prunus SLFL1*, *Prunus SLFL2*, *Prunus SLFL3*, *Malus SFBB*, and *Petunia SLF* genes. Numbers below the branches represent posterior credibility values above 60. In grey are the reference sequences (*Prunus SFB*, *Prunus SLFL*, Pyreae *SFBB*s, and *Petunia SLF* genes). Analysis utilized ClustalW2 alignment method.
(TIF)

S5 Fig Bayesian phylogenetic tree of the *P*. *persica and P*. *mume SFBB-* and *SFB-* like genes. The tree shows the relationship of *P*. *persica* (*ppa/ppb*) *and P*. *mume* (*P*. *mume scaffold*) *SFBB-* and *SFB-* like genes with *Prunus SFB*, *Prunus SLFL1*, *Prunus SLFL2*, *Prunus SLFL3*, *Malus SFBB*, and *Petunia SLF* genes. Numbers below the branches represent posterior credibility values above 60. In grey are the reference sequences (*Prunus SFB*, *Prunus SLFL*, Pyreae *SFBB*s, and *Petunia SLF* genes). Analysis utilized ClustalW2 alignment method.
(TIF)

S6 Fig Bayesian phylogenetic tree of the *F*. *vesca*, and *F. nipponica SFBB-* and *SFB-* like genes. The tree shows the relationship of the *F*. *vesca*, and *F*. *nipponica* F-box *SFBB-* and *SFB-* like genes with *Prunus SFB*, *Prunus SLFL1*, *Prunus SLFL2*, *Prunus SLFL3*, *Malus SFBB*, and *Petunia SLF* genes. Numbers below the branches represent posterior credibility values above 60. In grey are the reference sequences (*Prunus SFB*, *Prunus SLFL*, Pyreae *SFBB*s, and *Petunia SLF* genes). Analysis utilized ClustalW2 alignment method.
(TIF)

S7 Fig Bayesian phylogenies of Rosaceae *SFBB-* and *SFB-* like genes, using ClustalW2 (A), and Muscle (B). The trees show the relationship of *M*. *x domestica* (*MDP*), *P*. *persica* (*P*. *persica ppa*), *P*. *mume*, *F*. *vesca*, and *F*. *nipponica SFBB-* and *SFB-* like genes. Legend as in Fig 3.
(TIF)

S1 File FASTA file of the *M*. *× domestica*, *P*. *persica*, *P*. *mume*, *F*. *vesca*, and *F*. *nipponica T2-RNase* genes, larger than 500 bp, used in the phylogenetic analyses. (TXT)

S1 Table *M*. *fusca* RNA-seq data. (DOCX)

S2 Table *M*. *x domestica* (*MDP/MDC*) F-box genes, larger than 900 bp. (DOCX)

S3 Table *P*. *persica* (*P*. *persica ppa/ppb*) F-box genes, larger than 900 bp. (DOCX)

S4 Table *P*. *mume* F-box genes, larger than 900 bp. (DOCX)

S5 Table *F*. *vesca* F-box genes, larger than 900 bp. (DOCX)

S6 Table *F*. *nipponica* F-box genes, larger than 900 bp. (DOCX)

## References

[1] De Nettancourt D (1977) Incompatibility in angiosperms: Springer-Verlag, Berlin.

[2] Igic B, Lande R, Kohn JR (2008) Loss of self‐incompatibility and its evolutionary consequences. International Journal of Plant Sciences 169: 93–104.

[3] Roalson EH, McCubbin AG (2003) S-RNases and sexual incompatibility: structure, functions, and evolutionary perspectives. Molecular Phylogenetics and Evolution 29: 490–506. 14615188 10.1016/s1055-7903(03)00195-7

[4] Nowak MD, Davis AP, Anthony F, Yoder AD (2011) Expression and trans-specific polymorphism of self-incompatibility RNases in *Coffea* (Rubiaceae). PLoS One 6: e21019 10.1371/journal.pone.0021019 21731641 PMC3120821

[5] Igic B, Kohn JR (2001) Evolutionary relationships among self-incompatibility RNases. Proceedings of the National Academy of Sciences of the United States of America 98: 13167–13171. 11698683 10.1073/pnas.231386798 PMC60842

[6] Steinbachs JE, Holsinger KE (2002) *S*-RNase-mediated gametophytic self-incompatibility is ancestral in eudicots. Molecular Biology and Evolution 19: 825–829. 12032238 10.1093/oxfordjournals.molbev.a004139

[7] Vieira J, Fonseca NA, Vieira CP (2008) An *S-RNase*-based gametophytic self-incompatibility system evolved only once in eudicots. Journal of Molecular Evolution 67: 179–190. 10.1007/s00239-008-9137-x 18626680

[8] Igic B, Bohs L, Kohn JR (2006) Ancient polymorphism reveals unidirectional breeding system shifts. Proceedings of the National Academy of Sciences of the United States of America 103: 1359–1363. 16428289 10.1073/pnas.0506283103 PMC1360522

[9] Goldberg EE, Kohn JR, Lande R, Robertson KA, Smith SA, et al (2010) Species selection maintains self-incompatibility. Science 330: 493–495. 10.1126/science.1194513 20966249

[10] Igic B, Busch JW (2013) Is self‐fertilization an evolutionary dead end? New Phytologist 198: 386–397. 10.1111/nph.12182 23421594

[11] Ushijima K, Sassa H, Tamura M, Kusaba M, Tao R, Igić B (2001) Characterization of the S-locus region of almond (*Prunus dulcis*): analysis of a somaclonal mutant and a cosmid contig for an S haplotype. Genetics 158: 379–386. 11333246 10.1093/genetics/158.1.379 PMC1461623

[12] Ushijima K, Sassa H, Dandekar AM, Gradziel TM, Tao R, Hirano H (2003) Structural and transcriptional analysis of the self-incompatibility locus of almond: identification of a pollen-expressed F-box gene with haplotype-specific polymorphism. Plant Cell 15: 771–781. 12615948 10.1105/tpc.009290 PMC150029

[13] Entani T, Iwano M, Shiba H, Che FS, Isogai A, Takayama S (2003) Comparative analysis of the self-incompatibility (*S*-) locus region of *Prunus mume*: identification of a pollen-expressed F-box gene with allelic diversity. Genes to Cells 8: 203–213. 12622718 10.1046/j.1365-2443.2003.00626.x

[14] Ikeda K, Igic B, Ushijima K, Yamane H, Hauck N, Nakano R, et al (2004) Primary structural features of the *S* haplotype-specific F-box protein, SFB, in *Prunus* . Sexual Plant Reproduction 16: 235–243.

[15] Romero C, Vilanova S, Burgos L, Martinez-Calvo J, Vicente M, Llácer G, et al (2004) Analysis of the *S*-locus structure in *Prunus armeniaca* L. Identification of S-haplotype specific S-RNase and F-box genes. Plant Molecular Biology 56: 145–157. 15604734 10.1007/s11103-004-2651-3

[16] Sonneveld T, Tobutt KR, Vaughan SP, Robbins TP (2005) Loss of pollen-*S* function in two self-compatible selections of *Prunus avium* is associated with deletion/mutation of an *S* haplotype-specific F-box gene. Plant Cell 17: 37–51. 15598801 10.1105/tpc.104.026963 PMC544488

[17] Nunes MD, Santos RA, Ferreira SM, Vieira J, Vieira CP (2006) Variability patterns and positively selected sites at the gametophytic self-incompatibility pollen SFB gene in a wild self-incompatible *Prunus spinosa* (Rosaceae) population. New Phytologist 172: 577–587. 17083687 10.1111/j.1469-8137.2006.01838.x

[18] Vieira J, Santos RA, Ferreira SM, Vieira CP (2008) Inferences on the number and frequency of S-pollen gene (SFB) specificities in the polyploid *Prunus spinosa* . Heredity 101: 351–358. 10.1038/hdy.2008.60 18594559

[19] Cheng J, Han Z, Xu X, Li T (2006) Isolation and identification of the pollen-expressed polymorphic F-box genes linked to the *S*-locus in apple (*Malus* × *domestica*). Sexual Plant Reproduction 19: 175–183.

[20] Kakui H, Tsuzuki T, Koba T, Sassa H (2007) Polymorphism of SFBB-gamma and its use for *S* genotyping in Japanese pear (*Pyrus pyrifolia*). Plant Cell Reports 26: 1619–1625. 17541597 10.1007/s00299-007-0386-8

[21] Sassa H, Kakui H, Miyamoto M, Suzuki Y, Hanada T, Ushijima K, et al (2007) *S locus F-Box brothers*: multiple and pollen-specific F-box genes with *S* haplotype-specific polymorphisms in apple and Japanese pear. Genetics 175: 1869–1881. 17237509 10.1534/genetics.106.068858 PMC1855134

[22] Minamikawa M, Kakui H, Wang S, Kotoda N, Kikuchi S, Koba T, et al (2010) Apple *S* locus region represents a large cluster of related, polymorphic and pollen-specific F-box genes. Plant Molecular Biology 74: 143–154. 10.1007/s11103-010-9662-z 20628788

[23] Aguiar B, Vieira J, Cunha AE, Fonseca NA, Reboiro-Jato D, Reboiro-Jato M, et al (2013) Patterns of evolution at the gametophytic self-incompatibility *Sorbus aucuparia* (Pyrinae) *S* pollen genes support the non-self recognition by multiple factors model. Journal of Experimental Botany 64: 2423–2434. 10.1093/jxb/ert098 23606363 PMC3654429

[24] Tao R, Watari A, Hanada T, Habu T, Yaegaki H, Yamaguchi M, et al (2007) Self-compatible peach (*Prunus persica*) has mutant versions of the *S* haplotypes found in self-incompatible *Prunus* species. Plant molecular biology 63: 109–123. 17006593 10.1007/s11103-006-9076-0

[25] Yamane H, Ikeda K, Hauck NR, Iezzoni AF, Tao R (2003) Self-incompatibility (*S*) locus region of the mutated *S* ^6^-haplotype of sour cherry (*Prunus cerasus*) contains a functional pollen *S* allele and a non-functional pistil *S* allele. Journal of Experimental Botany 54: 2431–2437. 14512382 10.1093/jxb/erg271

[26] Kakui H, Kato M, Ushijima K, Kitaguchi M, Kato S, Sassa H (2011) Sequence divergence and loss-of-function phenotypes of *S locus F-box* brothers genes are consistent with non-self recognition by multiple pollen determinants in self-incompatibility of Japanese pear (*Pyrus pyrifolia*). Plant Journal 68: 1028–1038. 10.1111/j.1365-313X.2011.04752.x 21851432

[27] Okada K, Tonaka N, Taguchi T, Ichikawa T, Sawamura Y, Nakanishi T, et al (2011) Related polymorphic F-box protein genes between haplotypes clustering in the BAC contig sequences around the *S-RNase* of Japanese pear. Journal of Experimental Botany 62: 1887–1902. 10.1093/jxb/erq381 21172811 PMC3060677

[28] Wheeler D, Newbigin E (2007) Expression of 10 S-class *SLF-like* genes in *Nicotiana alata* pollen and its implications for understanding the pollen factor of the S locus. Genetics 177: 2171–2180. 17947432 10.1534/genetics.107.076885 PMC2219507

[29] Kubo K, Entani T, Takara A, Wang N, Fields AM, Hua Z, et al (2010) Collaborative non-self recognition system in S-RNase-based self-incompatibility. Science 330: 796–799. 10.1126/science.1195243 21051632

[30] Luu D-T, Qin X, Laublin G, Yang Q, Morse D, Cappadocia M (2001) Rejection of *S*-heteroallelic pollen by a dual-specific S-RNase in *Solanum chacoense* predicts a multimeric SI pollen xomponent. Genetics 159: 329–335. 11560908 10.1093/genetics/159.1.329 PMC1461794

[31] Hua Z, Kao TH (2006) Identification and characterization of components of a putative *Petunia S*-locus F-box-containing E3 ligase complex involved in S-RNase-based self-incompatibility. Plant Cell 18: 2531–2553. 17028207 10.1105/tpc.106.041061 PMC1626602

[32] Hua Z, Meng X, Kao TH (2007) Comparison of *Petunia inflata* S-Locus F-box protein (Pi SLF) with Pi SLF like proteins reveals its unique function in S-RNase based self-incompatibility. Plant Cell 19: 3593–3609. 18024566 10.1105/tpc.107.055426 PMC2174878

[33] Vieira J, Ferreira PG, Aguiar B, Fonseca NA, Vieira CP (2010) Evolutionary patterns at the RNase based gametophytic self—incompatibility system in two divergent Rosaceae groups (Maloideae and *Prunus*). BMC Evolutionary Biology 10: 200 10.1186/1471-2148-10-200 20584298 PMC2909234

[34] Vieira J, Fonseca NA, Vieira CP (2009) RNase-based gametophytic self-incompatibility evolution: Questioning the hypothesis of multiple independent recruitments of the S-pollen gene. Journal of Molecular Evolution 69: 32–41. 10.1007/s00239-009-9249-y 19495553

[35] Wang L, Dong L, Zhang Y, Zhang Y, Wu W, Deng X, et al (2004) Genome-wide analysis of *S*-Locus F-box-like genes in *Arabidopsis thaliana* . Plant Molecular Biology 56: 929–945. 15821991 10.1007/s11103-004-6236-y

[36] Ushijima K, Yamane H, Watari A, Kakehi E, Ikeda K, Hauck NR, et al (2004) The S haplotype-specific F-box protein gene, SFB, is defective in self-compatible haplotypes of *Prunus avium* and *P*. *mume* . Plant Journal 39: 573–586. 15272875 10.1111/j.1365-313X.2004.02154.x

[37] Vieira J, Morales-Hojas R, Santos RA, Vieira CP (2007) Different positively selected sites at the gametophytic self-incompatibility pistil S-RNase gene in the Solanaceae and Rosaceae (*Prunus*, *Pyrus*, and *Malus*). Journal of Molecular Evolution 65: 175–185. 17713808 10.1007/s00239-006-0285-6

[38] Matsumoto D, Yamane H, Tao R (2008) Characterization of *SLFL1*, a pollen-expressed F-box gene located in the *Prunus S* locus. Sexual Plant Reproduction 21: 113–121.

[39] Chantha S-C, Herman AC, Platts AE, Vekemans X, Schoen DJ (2013) Secondary evolution of a self-incompatibility locus in the Brassicaceae genus *Leavenworthia* . PLoS Biology 11: e1001560 10.1371/journal.pbio.1001560 23690750 PMC3653793

[40] Busch JW, Sharma J, Schoen DJ (2008) Molecular characterization of Lal2, an SRK-like gene linked to the S-locus in the wild mustard *Leavenworthia alabamica* . Genetics 178: 2055–2067. 10.1534/genetics.107.083204 18430933 PMC2323796

[41] Busch JW, Urban L (2011) Insights gained from 50 years of studying the evolution of self-compatibility in *Leavenworthia* (Brassicaceae). Evolutionary Biology 38: 15–27.

[42] Herman AC, Busch JW, Schoen DJ (2012) Phylogeny of *Leavenworthia S*-alleles suggests unidirectional mating system evolution and enhanced positive selection following an ancient population bottleneck. Evolution 66: 1849–1861. 10.1111/j.1558-5646.2011.01564.x 22671551

[43] Velasco R, Zharkikh A, Affourtit J, Dhingra A, Cestaro A, Kalyanaraman A, et al (2010) The genome of the domesticated apple (*Malus* x *domestica* Borkh.). Nature Genetics 42: 833–839. 10.1038/ng.654 20802477

[44] Verde I, Abbott AG, Scalabrin S, Jung S, Shu S, Marroni F, et al (2013) The high-quality draft genome of peach (*Prunus persica*) identifies unique patterns of genetic diversity, domestication and genome evolution. Nat Genet 45: 487–494. 10.1038/ng.2586 23525075

[45] Zhang Q, Chen W, Sun L, Zhao F, Huang B, Yang W, et al (2012) The genome of *Prunus mume* . Nature communications 3: 1318 10.1038/ncomms2290 23271652 PMC3535359

[46] Shulaev V, Sargent DJ, Crowhurst RN, Mockler TC, Folkerts O, Delcher AL, et al (2010) The genome of woodland strawberry (*Fragaria vesca*). Nature genetics 43: 109–116. 10.1038/ng.740 21186353 PMC3326587

[47] Hirakawa H, Shirasawa K, Kosugi S, Tashiro K, Nakayama S, Yamada M, et al (2013) Dissection of the octoploid strawberry genome by deep sequencing of the genomes of *Fragaria* species. DNA Research: 1–13. 10.1093/dnares/dst035 24282021 PMC3989489

[48] Njuguna W, Liston A, Cronn R, Ashman T-L, Bassil N (2012) Insights into phylogeny, sex function and age of *Fragaria* based on whole chloroplast genome sequencing. Molecular Phylogenetics and Evolution 66: 17–29. 10.1016/j.ympev.2012.08.026 22982444

[49] Bošković RI, Sargent DJ, Tobutt KR (2010) Genetic evidence that two independent S-loci control RNase-based self-incompatibility in diploid strawberry. Journal of experimental botany 61: 755–763. 10.1093/jxb/erp340 20008462 PMC2814107

[50] Rojas HJ, Roldán JA, Goldraij A (2013) NnSR1, a class III non-S-RNase constitutively expressed in styles, is induced in roots and stems under phosphate deficiency in *Nicotiana alata* . Annals of botany: 1–10. 10.1093/aob/mct244 24047716 PMC3806536

[51] Rice P, Longden I, Bleasby A (2000) EMBOSS: the European molecular biology open software suite. Trends in genetics 16: 276–277. 10827456 10.1016/s0168-9525(00)02024-2

[52] Vieira C, Charlesworth D (2002) Molecular variation at the self-incompatibility locus in natural populations of the genera *Antirrhinum* and *Misopates* . Heredity 88: 172–181. 11920118 10.1038/sj.hdy.6800024

[53] Artimo P, Jonnalagedda M, Arnold K, Baratin D, Csardi G, de Castro E, et al (2012) ExPASy: SIB bioinformatics resource portal. Nucleic acids research 40: W597–W603. 10.1093/nar/gks400 22661580 PMC3394269

[54] Reboiro-Jato D, Reboiro-Jato M, Fdez-Riverola F, Vieira CP, Fonseca NA, Vieira J (2012) ADOPS—Automatic Detection Of Positively Selected Sites. Journal of Integrative Bioinformatics 9: 200 10.2390/biecoll-jib-2012-200 22829571

[55] Huelsenbeck JP, Ronquist F (2001) MRBAYES: Bayesian inference of phylogenetic trees. Bioinformatics 17: 754–755. 11524383 10.1093/bioinformatics/17.8.754

[56] Blankenberg D, Kuster GV, Coraor N, Ananda G, Lazarus R, Mangan M, et al (2010) Galaxy: a web‐based genome analysis tool for experimentalists. Current protocols in molecular biology: 19.10.01–19.10.21. 20069535 10.1002/0471142727.mb1910s89 PMC4264107

[57] Giardine B, Riemer C, Hardison RC, Burhans R, Elnitski L, Shah P, et al (2005) Galaxy: a platform for interactive large-scale genome analysis. Genome research 15: 1451–1455. 16169926 10.1101/gr.4086505 PMC1240089

[58] Goecks J, Nekrutenko A, Taylor J (2010) Galaxy: a comprehensive approach for supporting accessible, reproducible, and transparent computational research in the life sciences. Genome Biol 11: R86 10.1186/gb-2010-11-8-r86 20738864 PMC2945788

[59] Haas BJ, Papanicolaou A, Yassour M, Grabherr M, Blood PD, Bowden J, et al (2013) De novo transcript sequence reconstruction from RNA-seq using the Trinity platform for reference generation and analysis. Nature protocols 8: 1494–1512. 10.1038/nprot.2013.084 23845962 PMC3875132

[60] Roberts A, Pachter L (2013) Streaming fragment assignment for real-time analysis of sequencing experiments. Nature methods 10: 71–73. 10.1038/nmeth.2251 23160280 PMC3880119

[61] Johnson M, Zaretskaya I, Raytselis Y, Merezhuk Y, McGinnis S, Madden TL (2008) NCBI BLAST: a better web interface. Nucleic acids research 36: W5–W9. 10.1093/nar/gkn201 18440982 PMC2447716

[62] Chagné D, Gasic K, Crowhurst RN, Han Y, Bassett HC, Bowatte DR, et al (2008) Development of a set of SNP markers present in expressed genes of the apple. Genomics 92: 353–358. 10.1016/j.ygeno.2008.07.008 18721872

[63] Habu T, Tao R (2014) Transcriptome Analysis of Self-and Cross-pollinated Pistils of Japanese Apricot (Prunus mume Sieb. et Zucc.). Journal of the Japanese Society for Horticultural Science 83: 95–107.

[64] Sun L, Zhang Q, Xu Z, Yang W, Guo Y, Lu J, et al (2013) Genome-wide DNA polymorphisms in two cultivars of mei (*Prunus mume* sieb. et zucc.). BMC genetics 14: 98 10.1186/1471-2156-14-98 24093913 PMC3851432

[65] Jung S, Cestaro A, Troggio M, Main D, Zheng P, Cho I, et al (2012) Whole genome comparisons of *Fragaria*, *Prunus* and *Malus* reveal different modes of evolution between Rosaceous subfamilies. BMC genomics 13: 1–12. 10.1186/1471-2164-13-1 22475018 PMC3368713

[66] Maliepaard C, Alston F, Van Arkel G, Brown L, Chevreau E, Dunemann F, et al (1998) Aligning male and female linkage maps of apple (*Malus pumila* Mill.) using multi-allelic markers. Theoretical and Applied Genetics 97: 60–73.

[67] Yamane H, Tao R, Mori H, Sugiura A (2003) Identification of a non-S RNase, a possible ancestral form of S-RNases, in *Prunus* . Molecular Genetics and Genomics 269: 90–100. 12715157 10.1007/s00438-003-0815-5

[68] Yamane H, Ushijima K, Sassa H, Tao R (2003) The use of the S haplotype-specific F-box protein gene, SFB, as a molecular marker for S-haplotypes and self-compatibility in Japanese apricot (*Prunus mume*). Theoretical and Applied Genetics 107: 1357–1361. 12920515 10.1007/s00122-003-1389-7

[69] Vilanova S, Badenes ML, Burgos L, Martínez-Calvo J, Llácer G, Romero C (2006) Self-compatibility of two apricot selections is associated with two pollen-part mutations of different nature. Plant Physiology 142: 629–641. 16920873 10.1104/pp.106.083865 PMC1586032

[70] Zuriaga E, Muñoz-Sanz JV, Molina L, Gisbert AD, Badenes ML, Romero C (2013) An *S*-Locus independent pollen factor confers self-compatibility in ‘Katy’ Apricot. PloS one 8: e53947 10.1371/journal.pone.0053947 23342044 PMC3544744

[71] Dirlewanger E, Graziano E, Joobeur T, Garriga-Calderé F, Cosson P, Howad W, et al (2004) Comparative mapping and marker-assisted selection in Rosaceae fruit crops. Proceedings of the National Academy of Sciences of the United States of America 101: 9891–9896. 15159547 10.1073/pnas.0307937101 PMC470769

[72] Illa E, Sargent DJ, Girona EL, Bushakra J, Cestaro A, Crowhurst R, et al (2011) Comparative analysis of rosaceous genomes and the reconstruction of a putative ancestral genome for the family. BMC Evolutionary biology 11: 9 10.1186/1471-2148-11-9 21226921 PMC3033827

[73] Evans W, Jones J (1967) Incompatibility in *Fragaria* . Canadian Journal of Genetics and Cytology 9: 831–836.

[74] Sargent DJ, Clarke J, Simpson D, Tobutt K, Arus P, Monfort A, et al (2006) An enhanced microsatellite map of diploid *Fragaria* . Theoretical and Applied Genetics 112: 1349–1359. 16505996 10.1007/s00122-006-0237-y

[75] Tao R, Iezzoni AF (2010) The S-RNase-based gametophytic self-incompatibility system in *Prunus* exhibits distinct genetic and molecular features. Scientia Horticulturae 124: 423–433.

[76] De Franceschi P, Dondini L, Sanzol J (2012) Molecular bases and evolutionary dynamics of self-incompatibility in the Pyrinae (Rosaceae). Journal of experimental botany 63: 4015–4032. 10.1093/jxb/ers108 22563122

[77] Sassa H, Kakui H, Minamikawa M (2010) Pollen-expressed F-box gene family and mechanism of S-RNase-based gametophytic self-incompatibility (GSI) in Rosaceae. Sexual Plant Reproduction 23: 39–43. 10.1007/s00497-009-0111-6 20165962

[78] Broothaerts W, Janssens GA, Proost P, Broekaert WF (1995) cDNA cloning and molecular analysis of two self-incompatibility alleles from apple. Plant molecular biology 27: 499–511. 7894015 10.1007/BF00019317

[79] Uyenoyama MK (2005) Evolution under tight linkage to mating type. New phytologist 165: 63–70. 15720621 10.1111/j.1469-8137.2004.01246.x

