# Supplementary figures and images for: Convergent Evolution at the Gametophytic Self-Incompatibility System in Malus and Prunus
Source: PLoS One. 2015 May 19;10(5):e0126138. doi: 10.1371/journal.pone.0126138 (PMC4438004; doi:10.1371/journal.pone.0126138)

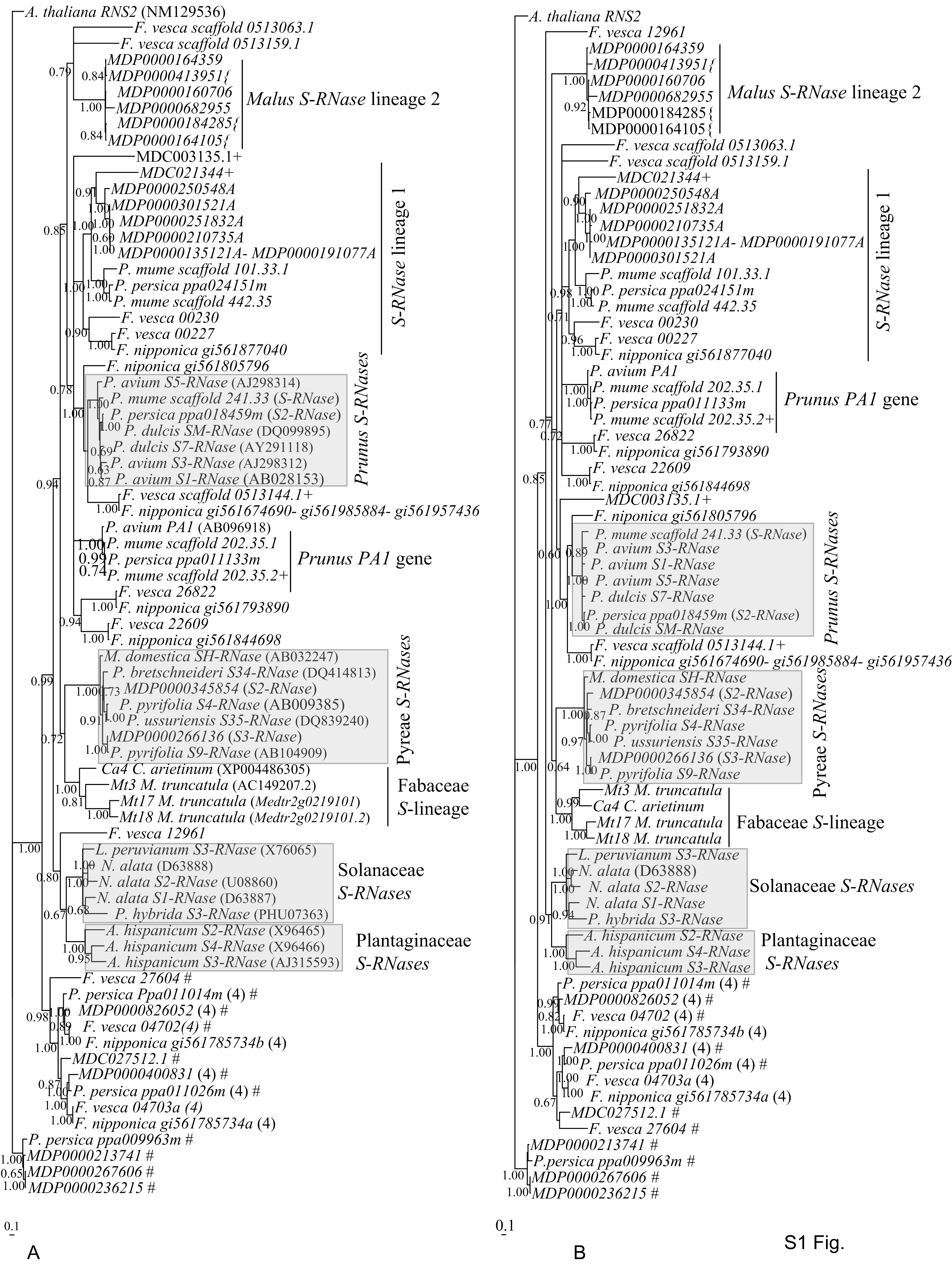

Supplement: S1 Fig — The trees show the relationship of the M. x domestica (MDP/MDC), P. persica (P. persica ppa/ppb), P. mume (P. mume scaffold), F. vesca, and F. nipponica T2-RNase lineage genes. Legend as in Fig 1. (TIF) [file pone.0126138.s001.tif]

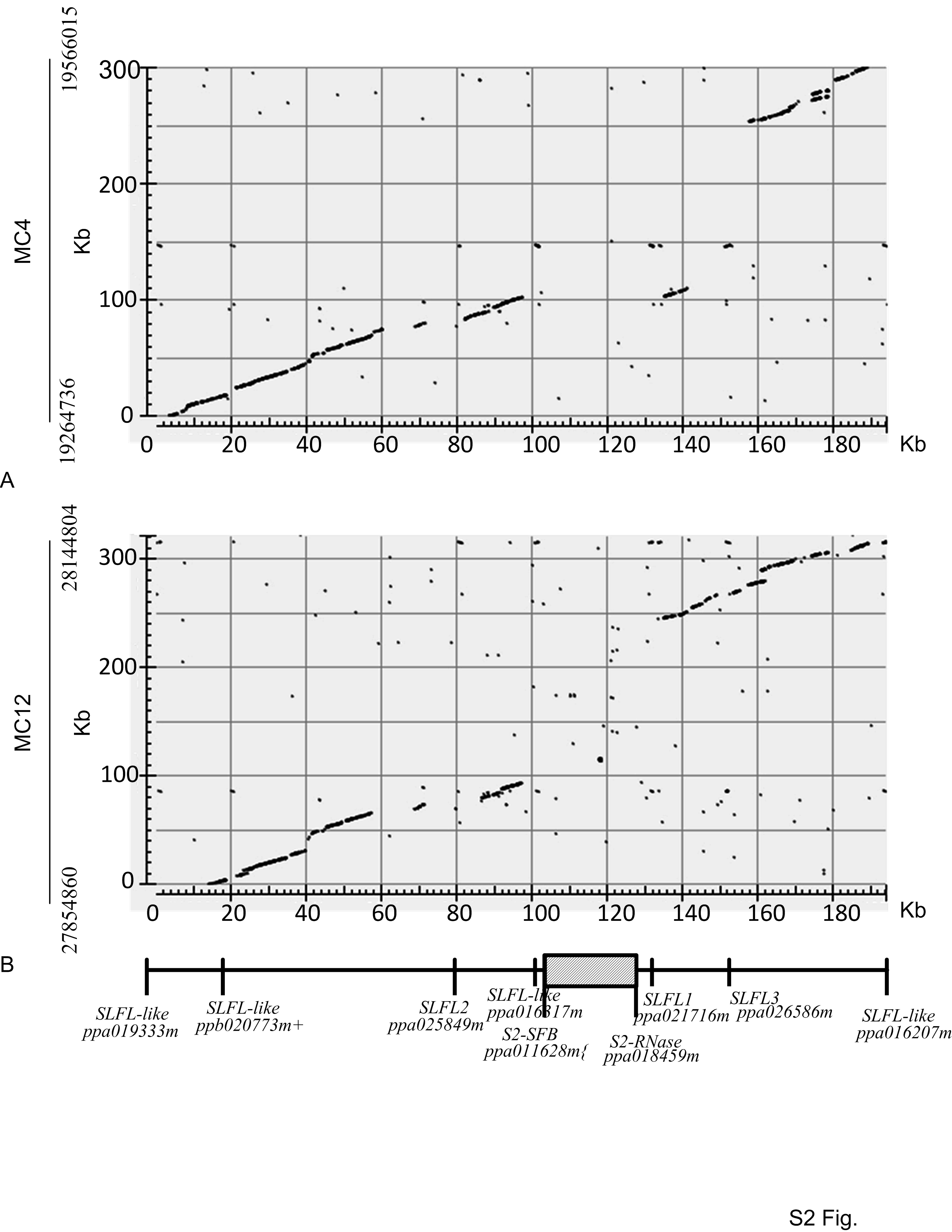

Supplement: S2 Fig — The Prunus region is that in between the ppa019333m (SLFL-like) and pp016207m (SLFL-like) (see S3 Table) while the Malus region is that in between position 19264736 to19566015 (MC4; panel A) and position 27854860 to 28144804 (MC12; panel B). (TIF) [file pone.0126138.s002.tif]

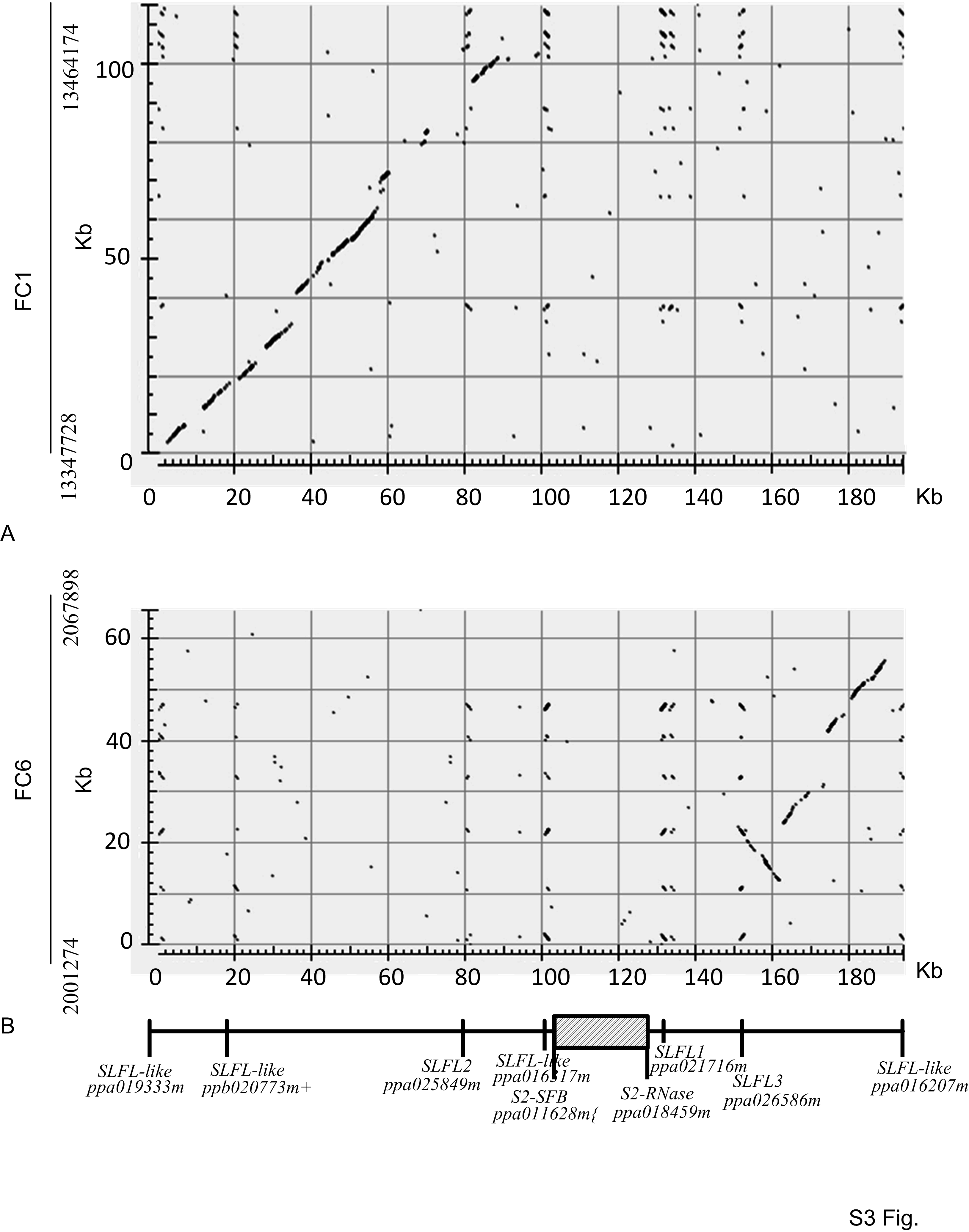

Supplement: S3 Fig — The Prunus region is that in between the ppa019333m (SLFL-like) and pp016207m (SLFL-like) (see S3 Table) while the Fragaria region is that in between position 13347728 to13464174 (FC1; panel A) and position 2001274 to 2067898 (FC6; panel B). (TIF) [file pone.0126138.s003.tif]

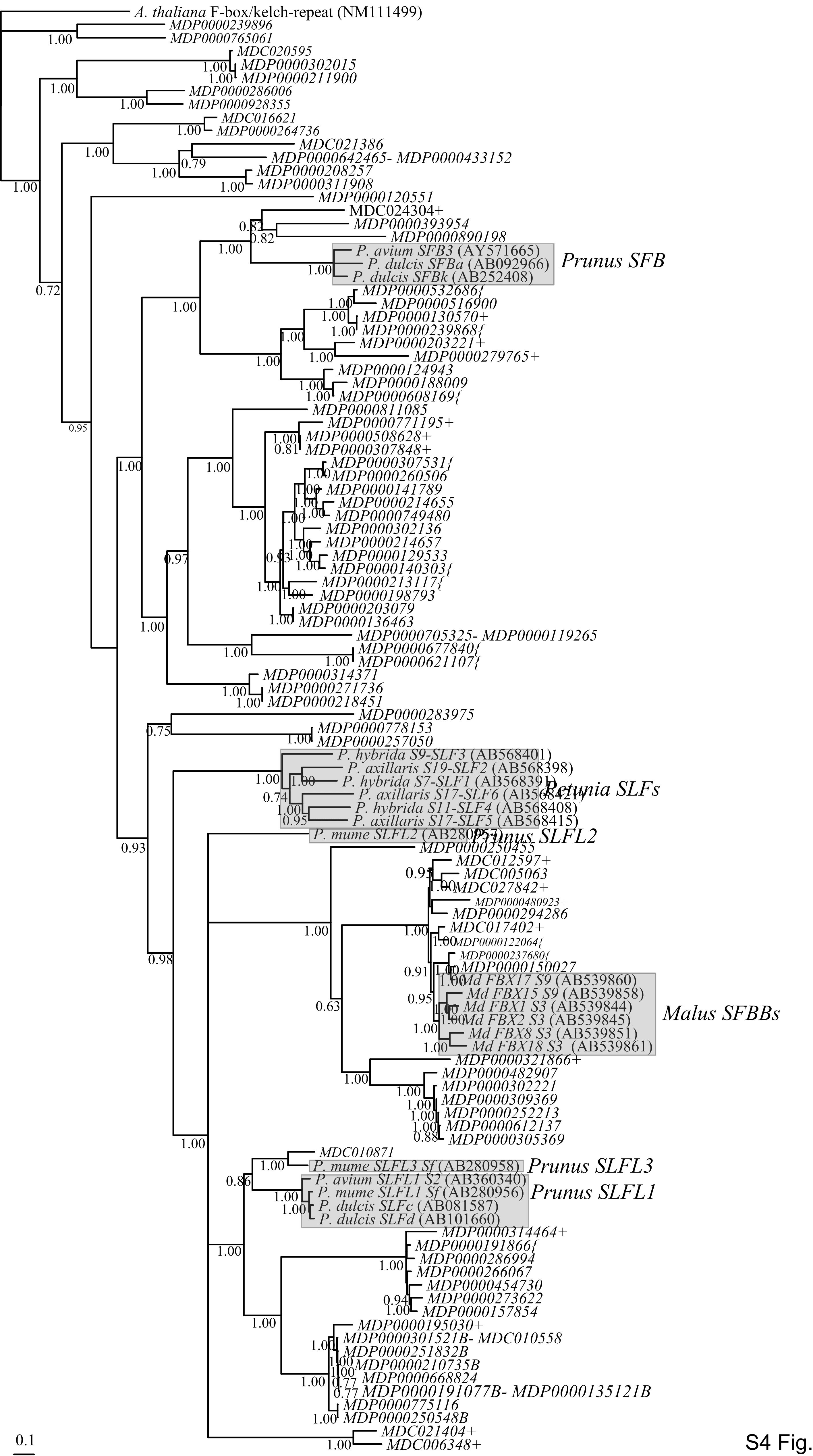

Supplement: S4 Fig — The tree shows the relationship of these genes with Prunus SFB, Prunus SLFL1, Prunus SLFL2, Prunus SLFL3, Malus SFBB, and Petunia SLF genes. Numbers below the branches represent posterior credibility values above 60. In grey are the reference sequences (Prunus SFB, Prunus SLFL, Pyreae SFBBs, and Petunia SLF genes). Analysis utilized ClustalW2 alignment method. (TIF) [file pone.0126138.s004.tif]

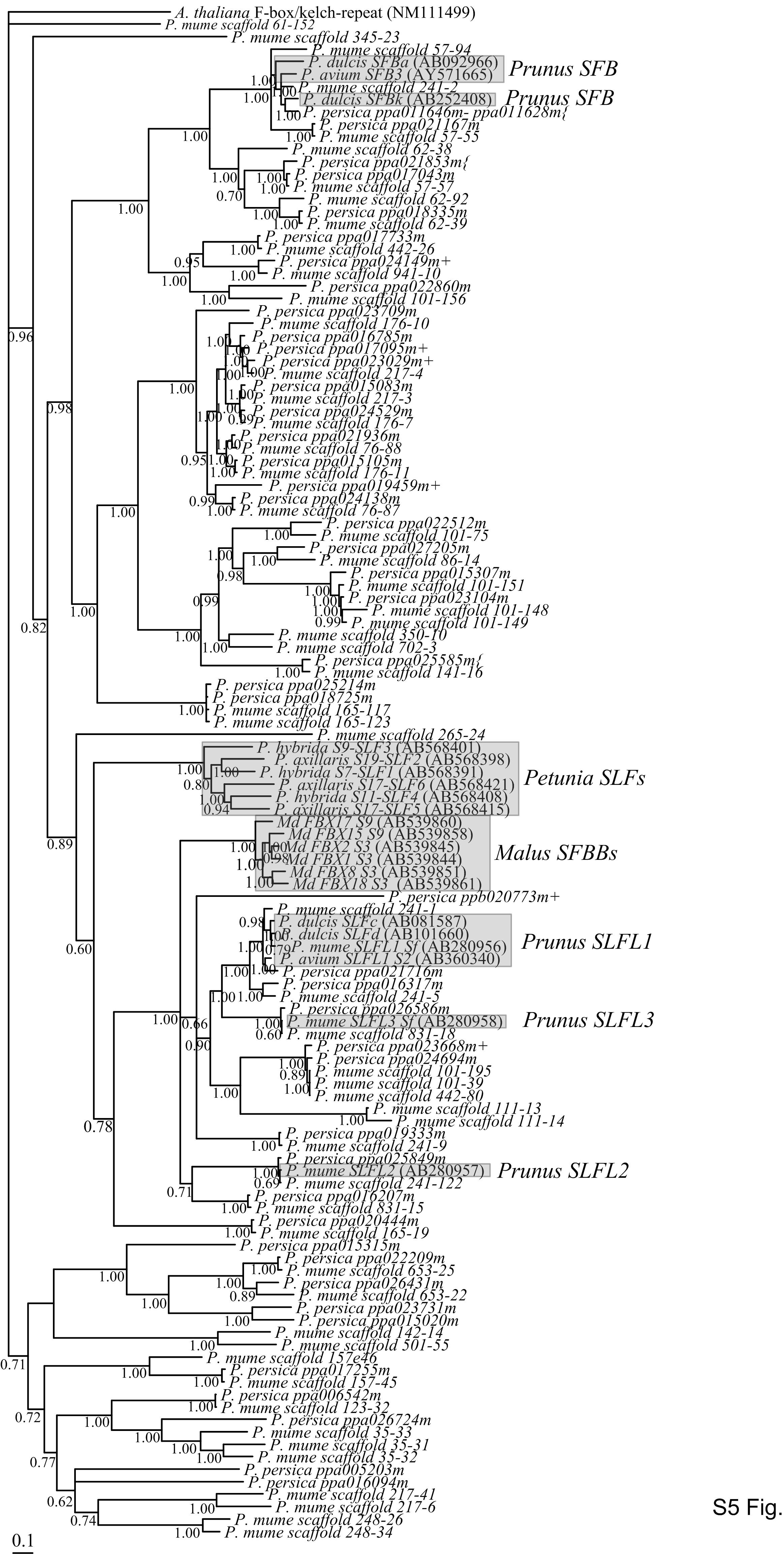

Supplement: S5 Fig — The tree shows the relationship of P. persica (ppa/ppb) and P. mume (P. mume scaffold) SFBB- and SFB- like genes with Prunus SFB, Prunus SLFL1, Prunus SLFL2, Prunus SLFL3, Malus SFBB, and Petunia SLF genes. Numbers below the branches represent posterior credibility values above 60. In grey are the reference sequences (Prunus SFB, Prunus SLFL, Pyreae SFBBs, and Petunia SLF genes). Analysis utilized ClustalW2 alignment method. (TIF) [file pone.0126138.s005.tif]

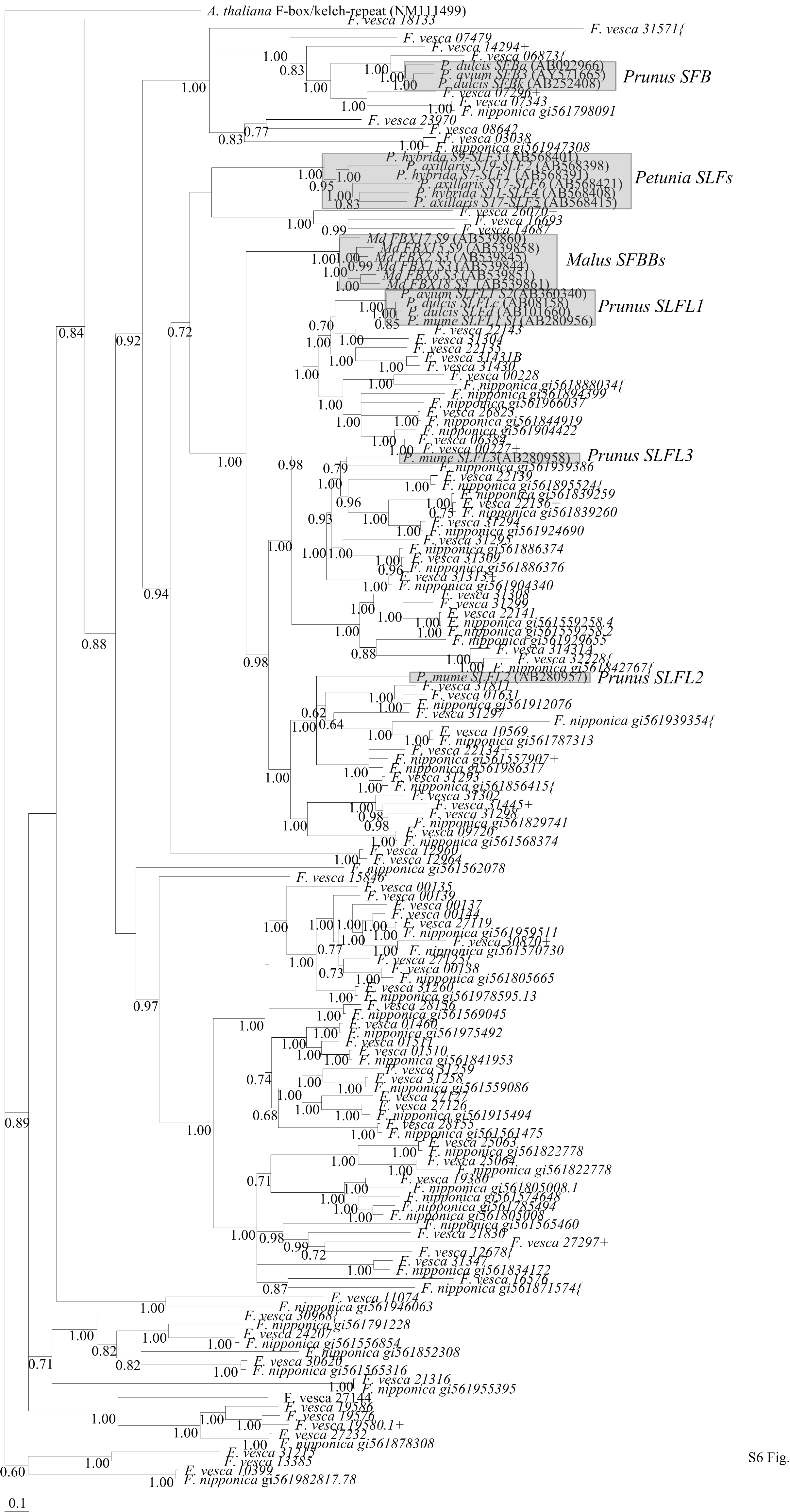

Supplement: S6 Fig — The tree shows the relationship of the F. vesca, and F. nipponica F-box SFBB- and SFB- like genes with Prunus SFB, Prunus SLFL1, Prunus SLFL2, Prunus SLFL3, Malus SFBB, and Petunia SLF genes. Numbers below the branches represent posterior credibility values above 60. In grey are the reference sequences (Prunus SFB, Prunus SLFL, Pyreae SFBBs, and Petunia SLF genes). Analysis utilized ClustalW2 alignment method. (TIF) [file pone.0126138.s006.tif]

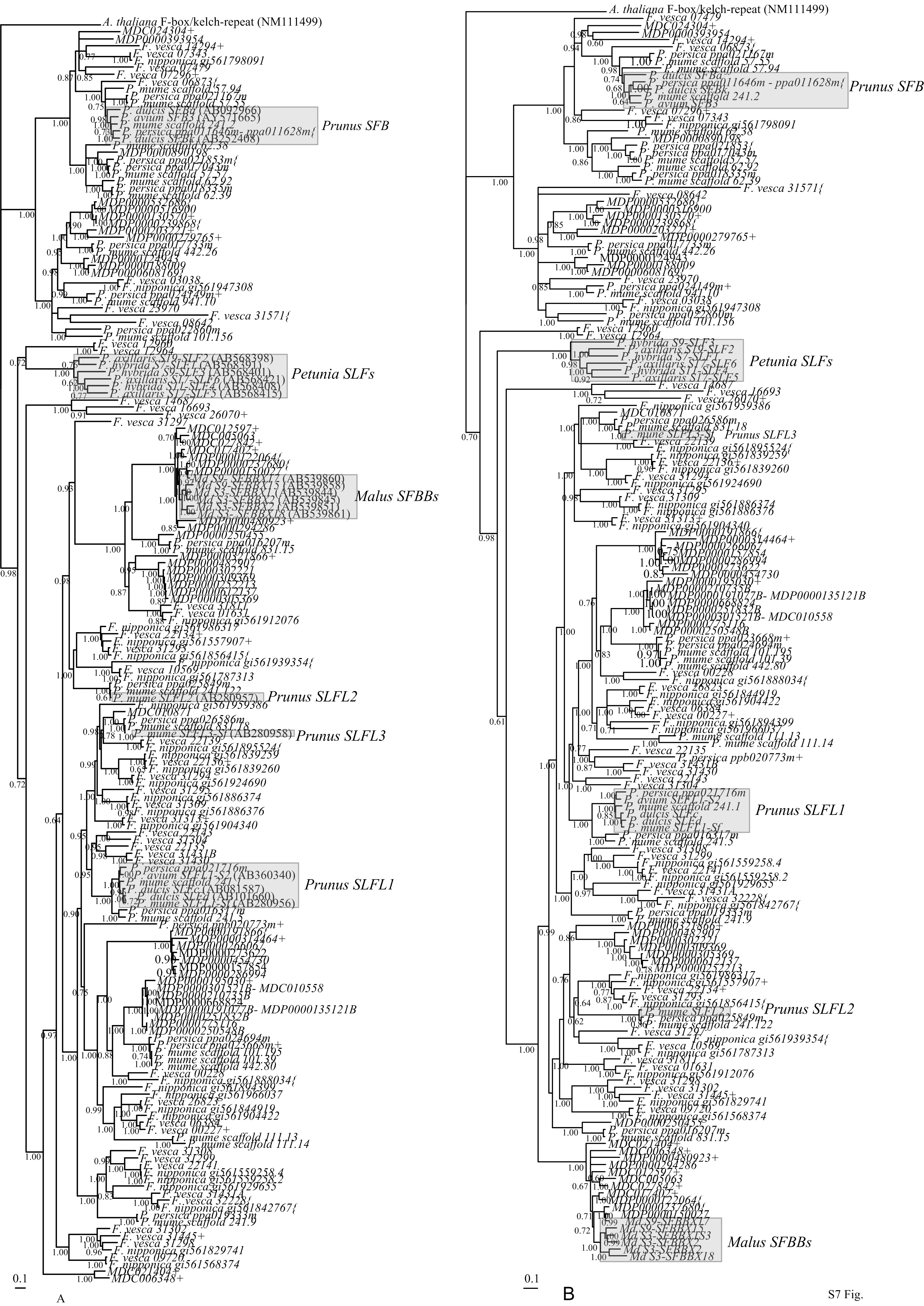

Supplement: S7 Fig — The trees show the relationship of M. x domestica (MDP), P. persica (P. persica ppa), P. mume, F. vesca, and F. nipponica SFBB- and SFB- like genes. Legend as in Fig 3. (TIF) [file pone.0126138.s007.tif]
