# Supplementary material for: Convergent Evolution at the Gametophytic Self-Incompatibility System in Malus and Prunus
Source: PLoS One. 2015 May 19;10(5):e0126138. doi: 10.1371/journal.pone.0126138 (PMC4438004; doi:10.1371/journal.pone.0126138)
Supplement: S1 Table — (DOCX) [file pone.0126138.s009.docx]

**Table S1**. *M. fusca* RNA-seq data

| **Description** | **Data Set** | **# Reads** | **% of >= Q30 Bases** | **Mean Quality** |
| --- | --- | --- | --- | --- |
| Entire flower buds not including pedicels; petals emerging | R9_1 | 30,621,152 | *nd* | *nd* |
|  | R9_2 | 34,308,486 | 80 | 32.28 |
|  | R9_3 | 19,752,388 | 85.22 | 33.87 |
| Flower pedicels | R10_1 | 26,497,798 | *nd* | *nd* |
|  | R10_2 | 27,956,018 | 79.73 | 32.19 |
|  | R10_3 | 17,588,352 | 85.75 | 34.05 |
| Mostly or fully expanded leaves from several nodes along stem including vegetative nodes near the base and flowering nodes closer to the apex | R12_1 | 28,099,080 | 78.27 | 31.81 |
|  | R12_2 | 33,070,014 | 80.4 | 32.41 |
|  | R12_3 | 18,183,332 | 86.38 | 34.26 |
| Petioles from expanded leaves from several nodes along stem including vegetative nodes near the base and flowering nodes closer to the apex | R14_1 | 18,912,840 | 79.25 | 32.11 |
|  | R14_2 | 37,530,824 | 80.42 | 32.41 |
|  | R14_3 | 18,420,518 | 85.35 | 33.9 |
| Flower petals ~1 week prior to anthesis1 | R15_1 | 24,556,472 | 80.25 | 32.37 |
|  | R15_2 | 19,111,692 | 75.74 | 31.2 |
|  | R15_3 | 18,880,598 | 85.68 | 34.03 |
| Flower pistils ~1 week prior to anthesis | R16_1 | 47,216,126 | 80.41 | 32.41 |
|  | R16_2 | 27,022,958 | 73.91 | 30.65 |
|  | R16_3 | 15,808,380 | 84.29 | 33.55 |
| Flower sepals ~1 week prior to anthesis | R18_1 | 24,436,462 | 79.57 | 32.14 |
|  | R18_2 | 18,704,416 | 74.9 | 30.95 |
|  | R18_3 | 17,645,202 | 85.55 | 34.01 |
| Flower stamens ~1 week prior to anthesis | R19_1 | 37,117,246 | 77.89 | 31.69 |
|  | R19_2 | 20,223,670 | 74.61 | 30.85 |
|  | R19_3 | 16,694,774 | 86.48 | 34.28 |
| Anthers at anthesis | R23_2 | 36,587,052 | 77.42 | 31.52 |
|  | R23_3 | 17,570,958 | 84.97 | 33.79 |
| Stamen filaments at anthesis | R25_1 | 33,749,220 | 77.99 | 31.71 |
|  | R25_3 | 16,352,486 | 85.79 | 34.02 |
| Basal section of pistils at anthesis | R26_1 | 28,401,362 | 75.41 | 31.1 |
|  | R26_2 | 47,011,298 | 84.41 | 33.56 |
|  | R26_3 | 19,885,086 | 85.73 | 34.04 |
| Pollen at anthesis | R28_2 | 22,983,314 | 80.05 | 32.36 |
|  | R28_3 | 18,303,100 | 84.74 | 33.74 |
| Stigmas from flowers at anthesis | R31_1 | 24,950,678 | 81.15 | 32.73 |
|  | R31_3 | 14,019,952 | 83.55 | 33.32 |
| Styles from flowers at anthesis | R32_3 | 16,872,202 | 85.31 | 33.92 |
| Fruit minus seeds ~1 week after anthesis | R35_1 | 19,231,528 | 81.22 | 32.73 |
|  | R35_3 | 19,326,736 | 84.94 | 33.82 |
| Fertilized ovules ~1 week after anthesis | R36_3 | 25,912,720 | 85.37 | 33.96 |
| Embryo dissected from seed 1 mm or less in diameter ~3 weeks after anthesis | R39_3 | 18,243,256 | 85.97 | 34.12 |
