# Supplementary material for: Convergent Evolution at the Gametophytic Self-Incompatibility System in Malus and Prunus
Source: PLoS One. 2015 May 19;10(5):e0126138. doi: 10.1371/journal.pone.0126138 (PMC4438004; doi:10.1371/journal.pone.0126138)
Supplement: S2 Table — (DOCX) [file pone.0126138.s010.docx]

**Table S2**. *M. domestica* (*MDP/MDC*) F-box genes, larger than 900 bp, obtained using as query *M. domestica* *SFBB3-beta* (AB270796) and *P. avium S3-SFB* (AY571665) protein sequences without the F-box region, and a expect value lower than *e*-12.

| Gene & | Location |
| --- | --- |
| *MDP0000195030* | chr1:15112330..15113770 |
| *MDC012597*+ | chr2:4100397..4101612 |
| *MDP0000928355* | chr2:8890996..8892049 |
| *MDP0000208257* | chr2:30132849..30133986 |
| *MDP0000311908* | chr2:30146864..30148010 |
| *MDP0000141789* | chr3:2447275..2448187 |
| *MDP0000302136* | chr3:2457298..2458549 |
| *MDP0000213117*{ | chr3:2586454..2587653 |
| *MDP0000705325- MDP0000119265* | chr4:7330203..7331388 |
| *MDC010871* | chr4:19506886..19508158 |
| *MDC016621* | chr5:495053..496187 |
| *MDP0000129533* | chr5:773685..774897 |
| *MDP0000811085* | chr5:12171759..12173001 |
| *MDP0000677840*{ | chr8:13866821..13868098 |
| *MDP0000621107*{ | chr8:13861064..13862339 |
| *MDP0000314464*+ | chr8:15408226..15409843 |
| *MDP0000252213* | chr9:9451048..9452287 |
| *MDP0000309369* | chr9:9472773..9473982 |
| *MDP0000612137* | chr9:9486121..9487360 |
| *MDP0000305369* | chr9:9499453..9500494 |
| *MDP0000188009* | chr9:30591575..30592514 |
| *MDP0000608169*{ | chr9:30607155..30608223 |
| *MDP0000532686*{ | chr9:30633626..30634682 |
| *MDP0000124943* | chr9:30659635..30660715 |
| *MDP0000239868*{ | chr9:30662090..30663281 |
| *MDP0000301521B- MDC010558* | chr10:16049269..16050748 |
| *MDP0000210735B* | chr10:16053480..16054863 |
| *MDP0000668824* | chr10:16056982..16058482 |
| *MDP0000251832B* | chr10:16072373..16073873 |
| *MDP0000191077B- MDP0000135121B* | chr10:16076829..16078329 |
| *MDP0000775116* | chr10:16172926..16174375 |
| *MDP0000286006* | chr10:20776979..20778137 |
| *MDP0000314371* | chr10:30988971..30990084 |
| *MDP0000264736* | chr10:32390589..32391723 |
| *MDP0000266067* | chr10:32536060..32537761 |
| *MDP0000136463* | chr10:33051994..33053203 |
| *MDP0000198793* | chr10:33064507..33065446 |
| *MDP0000140303*{ | chr10:33120909..33122116 |
| *MDP0000214655* | chr10:33122395..33123592 |
| *MDP0000307531*{ | chr10:33125628..33126839 |
| *MDP0000214657* | chr10:33129276..33130470 |
| *MDP0000260506* | chr10:33131903..33133115 |
| *MDP0000749480* | chr10:33135433..33136675 |
| *MDP0000508628+* | chr11:3053749..3054914 |
| *MDP0000307848*+ | chr11:3082452..3083721 |
| *MDP0000771195*+ | chr11:3087523..3088977 |
| *MDC021386* | chr11:4995537..4996722 |
| *MDP0000191866*{ | chr11:7799015..7799926 |
| *MDP0000286994* | chr11:7803806..7805453 |
| *MDP0000765061* | chr11:21452714..21453854 |
| *MDP0000239896* | chr11:21474958..21476098 |
| *MDP0000279765*+ | chr12:4963575..4964636 |
| *MDP0000250455* | chr12:28169466..28170633 |
| *MDP0000120551* | chr12:28692416..28693757 |
| *MDP0000890198* | chr13:29605654..29606770 |
| *MDC024304*+ | chr13:29610726..29611840 |
| *MDP0000393954* | chr13:29647634..29648702 |
| *MDP0000157854* | chr13:31337845..31339459 |
| *MDP0000203221*+ | chr13:32778100..32779174 |
| *MDP0000203079* | chr14:28562538..28563741 |
| *MDC020595* | chr15:7582350..7583778 |
| *MDP0000302015* | chr15:7618694..7620104 |
| *MDP0000211900* | chr15:7618807..7620217 |
| *MDP0000482907* | chr15:8210567..8211821 |
| *MDP0000454730* | chr15:9332590..9334279 |
| *MDP0000273622* | chr15:9639321..9641019 |
| *MDP0000150027* | chr16:5313986..5315168 |
| *MDP0000122064*{ | chr16:10808145..10809329 |
| *MDC017402*+ | chr16:20506662..20507845 |
| *MDP0000283975* | chr17:1425039..1426290 |
| *MDP0000642465- MDP0000433152* | chr17:8284871..8286212 |
| *MDP0000778153* | chr17:17437100..17438195 |
| *MDP0000257050* | chr17:17443211..17444306 |
| *MDP0000218451* | chr17:19431136..19432282 |
| *MDP0000271736* | chr17:19456973..19458119 |
| *MDC021404*+ | chr17:21266799..21267986 |
| *MDP0000294286* | chr17:21308644..21309829 |
| *MDC005063* | chr17:21370441..21371641 |
| *MDC006348*+ | chr17:21602540..21603662 |
| *MDC027842*+ | chr17:21633727..21634928 |
| *MDP0000237680*{ | chr17:21775307..21776490 |
| *MDP0000480923*+ | chr17:21910339..21911540 |
| *MDP0000321866*+ | unanchored:12710745..12711738 |
| *MDP0000250548B* | unanchored:63258661..63260110 |
| *MDP0000302221* | unanchored:95487681..95488692 |
| *MDP0000130570*+ | unanchored:101783013..101784348 |
| *MDP0000516900* | unanchored:109065168..109066164 |

&- it should be noted that alternative human-curated gene annotations have been used for these genes.

+ stop codons are found in the sequence

{ gaps were introduced to avoid stop codons
