# Supplementary material for: Convergent Evolution at the Gametophytic Self-Incompatibility System in Malus and Prunus
Source: PLoS One. 2015 May 19;10(5):e0126138. doi: 10.1371/journal.pone.0126138 (PMC4438004; doi:10.1371/journal.pone.0126138)
Supplement: S3 Table — (DOCX) [file pone.0126138.s011.docx]

**Table S3**. *P. persica* (*ppa/ppb*)F-box genes, larger than 900 bp, obtained using as query *Prunus SLFL1* (AB360342), *Malus SFBB3-beta* (AB270796) and *Prunus* *SFB* (AY571665) sequences without the F-box region, and a expect value lower than *e*-12

| Gene& | Location |  |  |
| --- | --- | --- | --- |
| *ppa022209m* | scaffold_1:11104442..11103366 |  |  |
| *ppa026431m* | scaffold_1:11107823..11106606 |  |  |
| *ppa025585m*{ | scaffold_1:13420455..13421647 |  |  |
| *ppa018335m* | scaffold_1:16265159..16264047 |  |  |
| *ppa024149m*+ | scaffold_2:15040308..15038781 |  |  |
| *ppa019459m*+ | scaffold_2:23217849..23219025 |  | |
| *ppa017733m* | scaffold_3:82421..83440 |  |  |
| *ppa022860m* | scaffold_3:88778..89716 |  |  |
| *ppa021167m* | scaffold_3:2482639..2483766 |  |  |
| *ppa017043m* | scaffold_3:2500078..2501214 |  |  |
| *ppa021853m*{ | scaffold_3:2503710..2504805 |  |  |
| *ppa026724m* | scaffold_3:6190370..6191434 |  |  |
| *ppa020444m* | scaffold_3:8117500..8116370 |  |  |
| *ppa025214m* | scaffold_3:8216219..8217337 |  |  |
| *ppa018725m* | scaffold_3:8292968..8294116 |  |  |
| *ppa015020m* | scaffold_3:8459689..8460966 |  | |
| *ppa023731m* | scaffold_3:8530667..8531926 |  |  |
| *ppa017255m* | scaffold_3:15052303..15051131 |  |  |
| *ppa006542m* | scaffold_4:472603..473784 |  |  |
| *ppa015105m* | scaffold_4:8612480..8611293 |  |  |
| *ppa016785m* | scaffold_4:8626706..8625480 |  |  |
| *ppa024529m* | scaffold_4:8631673..8630453 |  |  |
| *ppa023029m*+ | scaffold_4:11314564..11313329 |  |  |
| *ppa015083m* | scaffold_4:13895017..13896237 |  |  |
| *ppa017095m*+ | scaffold_4:13899738..13900742 |  |  |
| *ppa021936m* | scaffold_5:9505535..9504363 |  |  |
| *ppa024138m* | scaffold_5:9509017..9507764 |  |  |
| *ppa023709m* | scaffold_6:6653434..6654705 |  |  |
| *ppa027205m* | scaffold_6:24733960..24732689 |  |  |
| *ppa019333m* | scaffold_6:26319994..26318630 |  |  |
| *ppb020773m*+ | scaffold_6:26339332..26338173 |  |  |
| *ppa025849m* | scaffold_6:26398922..26400193 |  |  |
| *ppa016317m* | scaffold_6:26420476..26419253 |  |  |
| *ppa011646m/ ppa011628m*{ | scaffold_6:26422392..26423524 |  |  |
| *ppa021716m* | scaffold_6:26450833..26449619 |  |  |
| *ppa026586m* | scaffold_6:26470157..26471368 |  |  |
| *ppa016207m* | scaffold_6:26513040..26511865 |  |  |
| *ppa016094m* | scaffold_7:3683808..3684974 |  | |
| *ppa005203m* | scaffold_7:22519699..22518284 |  |  |
| *ppa022512m* | scaffold_8:18819137..18817809 |  |  |
| *ppa015307m* | scaffold_8:18869498..18870712 |  |  |
| *ppa023104m* | scaffold_8:18872992..18874218 |  |  |
| *ppa023668m*+ | scaffold_8:19231108..19229803 |  |  |
| *ppa024694m* | scaffold_8:19239450..19240769 | In the vicinity of *ppa024151m* |  |
| *ppa015315m* | scaffold_15:92521..91346 |  | |

&- it should be noted that alternative human-curated gene annotations have been used for these genes.

+ stop codons are found in the sequence;

{ gapes were introduced to avoid stop codons
