# Supplementary material for: Convergent Evolution at the Gametophytic Self-Incompatibility System in Malus and Prunus
Source: PLoS One. 2015 May 19;10(5):e0126138. doi: 10.1371/journal.pone.0126138 (PMC4438004; doi:10.1371/journal.pone.0126138)
Supplement: S4 Table — (DOCX) [file pone.0126138.s012.docx]

**Table S4**. *P. mume* box genes, larger than 900 bp, obtained using as query *Prunus SLFL1* (AB360342), *Malus SFBB3-beta* (AB270796) and *Prunus* *SFB* (AY571665) sequences without the F-box region, and a expect value lower than *e*-12

| Gene | Location |
| --- | --- |
| *P. mume scaffold 101-148* | gb\|AOHF01009195.1\|: 2161.. 3365 |
| *P. mume scaffold 101-149* | gb\|AOHF01009199.1\|: 3872.. 5079 |
| *P. mume scaffold 101-151* | gb\|AOHF01009200.1\|: 10175.. 11407 |
| *P. mume scaffold 101-156* | gb\|AOHF01009206.1\|: 1491.. 2636 |
| *P. mume scaffold 101-195* | gb\|AOHF01009225.1\|: 4734.. 6059 |
| *P. mume scaffold 101-39* | gb\|AOHF01009221.1\|: 89.. 1180 |
| *P. mume scaffold 101-75* | gb\|AOHF01009191.1\|: 11446.. 12735 |
| *P. mume scaffold 111-13* | gb\|AOHF01006122.1\|: 53420.. 54835 |
| *P. mume scaffold 111-14* | gb\|AOHF01006122.1\|: 61055.. 61978 |
| *P. mume scaffold 123-32* | gb\|AOHF01005628.1\|: 12309.. 13493 |
| *P. mume scaffold 141-16* | gb\|AOHF01003463.1\|: 12383.. 13585 |
| *P. mume scaffold 142-14* | gb\|AOHF01002490.1\|: 92.. 1399 |
| *P. mume scaffold 157-45* | gb\|AOHF01006970.1\|: 17793.. 18968 |
| *P. mume scaffold 157-46* | gb\|AOHF01006970.1\|: 15500.. 16759 |
| *P. mume scaffold 165-117* | gb\|AOHF01006322.1\|: 13410.. 14540 |
| *P. mume scaffold 165-123* | gb\|AOHF01006327.1\|: 1428.. 2567 |
| *P. mume scaffold 165-19* | gb\|AOHF01006312.1\|: 26017.. 27150 |
| *P. mume scaffold 176-10* | gb\|AOHF01004826.1\|: 17142.. 18488 |
| *P. mume scaffold 176-11* | gb\|AOHF01004826.1\|: 21765.. 23009 |
| *P. mume scaffold 176-7* | gb\|AOHF01004826.1\|: 5024.. 6301 |
| *P. mume scaffold 217-3* | gb\|AOHF01005136.1\|: 5638.. 6915 |
| *P. mume scaffold 217-4* | gb\|AOHF01005136.1\|: 9471.. 10643 |
| *P. mume scaffold 217-41* | gb\|AOHF01005143.1\|: 22159.. 23388 |
| *P. mume scaffold 217-6* | gb\|AOHF01005136.1\|: 42147.. 43421 |
| *P. mume scaffold 241-1* | gb\|AOHF01001494.1\|: 9551.. 10789 |
| *P. mume scaffold 241-122* | gb\|AOHF01001496.1\|: 17039.. 18325 |
| *P. mume scaffold 241-2* | gb\|AOHF01001494.1\|: 25169.. 26311 |
| *P. mume scaffold 241-5* | gb\|AOHF01001495.1\|: 25081.. 26382 |
| *P. mume scaffold 241-9* | gb\|AOHF01001500.1\|: 36023.. 37390 |
| *P. mume scaffold 248-26* | gb\|AOHF01004780.1\|: 5024.. 6193 |
| *P. mume scaffold 248-34* | gb\|AOHF01004784.1\|: 2742.. 3782 |
| *P. mume scaffold 265-24* | gb\|AOHF01013594.1\|: 46063.. 47259 |
| *P. mume scaffold 345-23* | gb\|AOHF01006994.1\|: 35809.. 37062 |
| *P. mume scaffold 35-31* | gb\|AOHF01004181.1\|: 19741.. 20997 |
| *P. mume scaffold 35-32* | gb\|AOHF01004181.1\|: 17139.. 18335 |
| *P. mume scaffold 35-33* | gb\|AOHF01004181.1\|: 14125.. 15279 |
| *P. mume scaffold 350-10* | gb\|AOHF01005117.1\|: 15903.. 17096 |
| *P. mume scaffold 442-26* | gb\|AOHF01005968.1\|: 9379.. 10506 |
| *P. mume scaffold 442-80* | gb\|AOHF01006022.1\|: 1447.. 2772 |
| *P. mume scaffold 501-55* | gb\|AOHF01000286.1\|: 7485.. 8675 |
| *P. mume scaffold 57-55* | gb\|AOHF01006065.1\|: 60631.. 61761 |
| *P. mume scaffold 57-57* | gb\|AOHF01006066.1\|: 11868.. 13007 |
| *P. mume scaffold 57-94* | gb\|AOHF01006065.1\|: 63402.. 64538 |
| *P. mume scaffold 61-152* | gb\|AOHF01008883.1\|: 2652.. 4061 |
| *P. mume scaffold 62-38* | gb\|AOHF01003275.1\|: 19283.. 20404 |
| *P. mume scaffold 62-39* | gb\|AOHF01003275.1\|: 14479.. 15594 |
| *P. mume scaffold 62-92* | gb\|AOHF01003307.1\|: 2581.. 3735 |
| *P. mume scaffold 653-22* | gb\|AOHF01003632.1\|: 48296.. 49444 |
| *P. mume scaffold 653-25* | gb\|AOHF01003632.1\|: 63960.. 65234 |
| *P. mume scaffold 702-3* | gb\|AOHF01004855.1\|: 3036.. 4211 |
| *P. mume scaffold 76-87* | gb\|AOHF01010842.1\|: 61136.. 62506 |
| *P. mume scaffold 76-88* | gb\|AOHF01010842.1\|: 57401.. 58630 |
| *P. mume scaffold 831-15* | gb\|AOHF01001551.1\|: 3243.. 4421 |
| *P. mume scaffold 831-18* | gb\|AOHF01001553.1\|: 34396.. 35619 |
| *P. mume scaffold 86-14* | gb\|AOHF01001442.1\|: 14505.. 15479 |
| *P. mume scaffold 941-10* | gb\|AOHF01008419.1\|: 1189.. 2283 |

+ stop codons are found in the sequence;

{ gapes were introduced to avoid stop codons
