# Supplementary material for: Convergent Evolution at the Gametophytic Self-Incompatibility System in Malus and Prunus
Source: PLoS One. 2015 May 19;10(5):e0126138. doi: 10.1371/journal.pone.0126138 (PMC4438004; doi:10.1371/journal.pone.0126138)
Supplement: S5 Table — (DOCX) [file pone.0126138.s013.docx]

**Table S5**: *F. vesca* F-box genes, larger than 900 bp, obtained using as query *Malus SFBB3-beta* (AB270796) and *Prunus SFB3* (AY571665) sequences without the F-box region as query, and a expect value lower than *e*-12

| Gene & | Location |
| --- | --- |
| *F. vesca 23970* | LG1:scf0512991 [1456057 - 1457214] |
| *F. vesca 31571*{ | LG1:scf0513096 [213798 - 213112] |
| *F. vesca 30870*+ | LG1:scf0513105 [443007 - 442141] |
| *F. vesca 12960* | LG1:scf0513192 [1520910 - 1522238] |
| *F. vesca 12964* | LG1:scf0513192 [1543944 - 1545278] |
| *F. vesca 14687* | LG1:scf0513192 [3176531 - 3175440] |
| *F. vesca 30968*{ | LG1:scf0513105 [886608 - 889190] |
| *F. vesca 12678*{ | LG1:scf0513192 [482960 - 485343] |
| *F. vesca 01631* | LG2:scf0512984 [287494 - 288411] |
| *F. vesca 31811* | LG2:scf0513097 [241772 - 242860] |
| *F. vesca 08642* | LG2:scf0513134 [999398 - 1000417] |
| *F. vesca 07343* | LG2:scf0513137 [270254 - 268992] |
| *F. vesca 10569* | LG2:scf0513144 [222294 - 223556] |
| *F. vesca 11074* | LG2:scf0513144 [624804 - 625997] |
| *F. vesca 00135*{ | LG3:scf0512941 [18997 - 18143] |
| *F. vesca 00137* | LG3:scf0512941 [26961 - 25699] |
| *F. vesca 00138* | LG3:scf0512941 [34887 - 33601] |
| *F. vesca 00139* | LG3:scf0512941 [39420 - 38131] |
| *F. vesca 00144* | LG3:scf0512941 [55803 - 54541] |
| *F. vesca 01460* | LG3:scf0512978 [511167 - 512378] |
| *F. vesca 01510* | LG3:scf0512978 [812155 - 813468] |
| *F. vesca 01511* | LG3:scf0512978 [816010 - 817251] |
| *F. vesca 30620* | LG3:scf0513104 [1326400 - 1327533] |
| *F. vesca 28155* | LG3:scf0513125 [1303634 - 1304866] |
| *F. vesca 28156* | LG3:scf0513125 [1305981 - 1307297] |
| *F. vesca 27119* | LG3:scf0513130 [9704 - 10987] |
| *F. vesca 27125*{ | LG3:scf0513130 [39302 - 40021] |
| *F. vesca 27126* | LG3:scf0513130 [46604 - 47803] |
| *F. vesca 27127* | LG3:scf0513130 [61325 - 62485] |
| *F. vesca 27232* | LG3:scf0513130 [824173 - 833553] |
| *F. vesca 27144* | LG3:scf0513130 [155111 - 156301] |
| *F. vesca 27297*+ | LG3:scf0513130 [1229904 - 1229293] |
| *F. vesca 10399* | LG3:scf0513142 [87233 - 86043] |
| *F. vesca 19576* | LG3:scf0513171 [398725 - 397385] |
| *F. vesca 19580*+ | LG3:scf0513171 [421458 - 422354] |
| *F. vesca 19586* | LG3:scf0513171 [445747 - 444542] |
| *F. vesca 06384* | LG4:scf0513158.5 [126057 - 127427] |
| *F. vesca 25063* | LG5:scf0513024 [268613 - 269782] |
| *F. vesca 25064* | LG5:scf0513024 [271254 - 272351] |
| *F. vesca 32228*{ | LG5:scf0513098 [637820 - 639342] |
| *F. vesca 31215* | LG5:scf0513106 [124310 - 125473] |
| *F. vesca 31258* | LG5:scf0513106 [452134 - 450905] |
| *F. vesca 31259* | LG5:scf0513106 [455330 - 454188] |
| *F. vesca 31260* | LG5:scf0513106 [460158 - 458881] |
| *F. vesca 26070*+ | LG5:scf0513111 [1274694 - 1275623] |
| *F. vesca 26823* | LG5:scf0513128 [356326 - 355007] |
| *F. vesca 00227*+ | LG6:scf0512945 [89412 - 100597] |
| *F. vesca 00228* | LG6:scf0512945 [102249 - 103559] |
| *F. vesca 24207* | LG6:scf0512999 [117228 - 118340] |
| *F. vesca 22134*+ | LG6:scf0513061 [16689 - 17636] |
| *F. vesca 22135* | LG6:scf0513061 [18294 - 19508] |
| *F. vesca 22136*+ | LG6:scf0513061 [28689 - 29294] |
| *F. vesca 22139* | LG6:scf0513061 [40112 - 38799] |
| *F. vesca 22141* | LG6:scf0513061 [49925 - 51271] |
| *F. vesca 22143* | LG6:scf0513061 [64601 - 63378] |
| *F. vesca 21830* | LG6:scf0513073 [231277 - 232458] |
| *F. vesca 31293* | LG6:scf0513095 [6138 - 4870] |
| *F. vesca 31294* | LG6:scf0513095 [8762 - 7515] |
| *F. vesca 31295* | LG6:scf0513095 [10934 - 9774] |
| *F. vesca 31299* | LG6:scf0513095 [24339 - 25643] |
| *F. vesca 31302* | LG6:scf0513095 [58150 - 56804] |
| *F. vesca 31304* | LG6:scf0513095 [63319 - 62084] |
| *F. vesca 31308* | LG6:scf0513095 [92370 - 93695] |
| *F. vesca 31309* | LG6:scf0513095 [101481 - 100240] |
| *F. vesca 31313*+ | LG6:scf0513095 [122355 - 122990] |
| *F. vesca 31297* | LG6:scf0513095 [145356 - 146603] |
| *F. vesca 31298* | LG6:scf0513095 [154220 - 155080] |
| *F. vesca 31430* | LG6:scf0513095 [165021 - 163786] |
| *F. vesca 31431B*{ | LG6:scf0513095 [170173 - 169400] |
| *F. vesca 31431A* | LG6:scf0513095 [173552 - 172317] |
| *F. vesca 31445*{ | LG6:scf0513095 [238383 - 240657] |
| *F. vesca 31347* | LG6:scf0513095 [651060 - 649870] |
| *F. vesca 09720* | LG6:scf0513149 [586513 - 587877] |
| *F. vesca 07296*+ | LG6:scf0513160 [2084139 - 2085272] |
| *F. vesca 07479* | LG6:scf0513160.6 [514689 - 513547] |
| *F. vesca 06873*{ | LG6:scf0513160.6 [114487 - 113804] |
| *F. vesca 15846* | LG6:scf0513165 [1788249 - 1787131] |
| *F. vesca 16576* | LG6:scf0513168.6 [180610 - 181866] |
| *F. vesca 16693* | LG6:scf0513168.6 [863363 - 864550] |
| *F. vesca 18133* | LG6:scf0513177 [170654 - 171757] |
| *F. vesca 21316* | LG7:scf0513044 [1566153 - 1564633] |
| *F. vesca 03038* | LG7:scf0513156 [246889 - 245801] |
| *F. vesca 19380* | LG7:scf0513170 [5091812 - 5090577] |
| *F. vesca 13385* | LG7:scf0513190 [3261416 - 3262555] |
| *F. vesca 14294*+ | LG7:scf0513179 [1320971 - 1321483] |

&- it should be noted that alternative human-curated gene annotations have been used for these genes.

+ stop codons are found in the sequence;

{ gapes were introduced to avoid stop codons
