# Supplementary material for: Convergent Evolution at the Gametophytic Self-Incompatibility System in Malus and Prunus
Source: PLoS One. 2015 May 19;10(5):e0126138. doi: 10.1371/journal.pone.0126138 (PMC4438004; doi:10.1371/journal.pone.0126138)
Supplement: S6 Table — (DOCX) [file pone.0126138.s014.docx]

**Table S6**: *F. nipponica* F-box genes, larger than 900 bp, obtained using as query *Malus SFBB3-beta* (AB270796) and *Prunus SFB3* (AY571665) sequences without the F-box region as query, and a expect value lower than *e*-12

| Gene | Contig |
| --- | --- |
| *F. nipponica gi561556854* | FNI_icon04474451.1: 887.. 1959 |
| *F. nipponica gi561557907+* | FNI_icon04467507.1.: 1151.. 2197 |
| *F. nipponica gi561559086* | FNI_icon04462005.1.: 21.. 1097 |
| *F. nipponica gi561559258.4* | FNI_icon04461197.1.: 62.. 1420 |
| *F. nipponica gi561559258.2* | FNI_icon04461197.1.: 116.. 1420 |
| *F. nipponica gi561561475* | FNI_icon04451229.1.: 1... 1171 |
| *F. nipponica gi561562078* | FNI_icon04449153.1.: 87... 1312 |
| *F. nipponica gi561565316* | FNI_icon04438109.1.; 333.. 1458 |
| *F. nipponica gi561565460* | FNI_icon04437663.1.: 137.. 1387 |
| *F. nipponica gi561568374* | FNI_icon04428611.1.: 41.. 1318 |
| *F. nipponica gi561569045* | FNI_icon04426539.1.: 1.. 1287 |
| *F. nipponica gi561570730* | FNI_icon04421799.1.: 217.. 1231 |
| *F. nipponica gi561574648* | FNI_icon04410529.1: 88.. 1119 |
| *F. nipponica gi561785494* | FNI_iscf00105449.1.: 1115.. 2702 |
| *F. nipponica gi561787313* | FNI_iscf00104447.1.: 650.. 1792 |
| *F. nipponica gi561791228* | FNI_iscf00102507.1.: 129.. 1241 |
| *F. nipponica gi561798091* | FNI_iscf00098619.1.: 13.. 1023 |
| *F. nipponica gi561805008* | FNI_iscf00094632.1.: 3305.. 4500 |
| *F. nipponica gi561805008.1* | FNI_iscf00094632.1.; 231.. 1496 |
| *F. nipponica gi561805665* | FNI_iscf00094253.1.: 315.. 1349 |
| *F. nipponica gi561822778* | FNI_iscf00084932.1.: 1702.. 2894 |
| *F. nipponica gi561822778* | FNI_iscf00084932.1.: 677.. 1672 |
| *F. nipponica gi561829741* | FNI_iscf00081382.1.: 482.. 1829 |
| *F. nipponica gi561834172* | FNI_iscf00079004.1.: 3.. 1109 |
| *F. nipponica gi561839259* | FNI_iscf00076273.1.: 1172.. 2419 |
| *F. nipponica gi561839260* | FNI_iscf00076272.1.: 564.. 1811 |
| *F. nipponica gi561841953* | FNI_iscf00074655.1.: 466.. 1782 |
| *F. nipponica gi561842767{* | FNI_iscf00074225.1.: 84.. 1804 |
| *F. nipponica gi561844919* | FNI_iscf00073088.1.: 1139.. 2442 |
| *F. nipponica gi561852308* | FNI_iscf00069194.1.: 81.. 1193 |
| *F. nipponica gi561856415{* | FNI_iscf00067034.1.: 737.. 1850 |
| *F. nipponica gi561871574{* | FNI_iscf00059300.1.: 648.. 1728 |
| *F. nipponica gi561878308* | FNI_iscf00055830.1.: 3841.. 2501 |
| *F. nipponica gi561886374* | FNI_iscf00051562.1.: 2.. 1069 |
| *F. nipponica gi561886376* | FNI_iscf00051561.1.: 2.. 1063 |
| *F. nipponica gi561888034{* | FNI_iscf00050728.1.: 451.. 1771 |
| *F. nipponica gi561894399* | FNI_iscf00047535.1.: 1.. 1218 |
| *F. nipponica gi561895524{* | FNI_iscf00046966.1.: 3254.. 4359 |
| *F. nipponica gi561904340* | FNI_iscf00042532.1.: 336.. 1634 |
| *F. nipponica gi561904422* | FNI_iscf00042489.1.: 1.. 986 |
| *F. nipponica gi561912076* | FNI_iscf00038878.1.:1668.. 2657 |
| *F. nipponica gi561915494* | FNI_iscf00037183.1.:1.. 979 |
| *F. nipponica gi561924690* | FNI_iscf00032413.1.:1644.. 2897 |
| *F. nipponica gi561929655* | FNI_iscf00029911.1.: 1621.. 2889 |
| *F. nipponica gi561939354{* | FNI_iscf00025048.1.: 350.. 1252 |
| *F. nipponica gi561946063* | FNI_iscf00021662.1.: 984.. 2198 |
| *F. nipponica gi561947308* | FNI_iscf00021032.1.: 719.. 1687 |
| *F. nipponica gi561955395* | FNI_iscf00017157.1.: 4970.. 6316 |
| *F. nipponica gi561959386* | FNI_iscf00015039.1.: 3591.. 4904 |
| *F. nipponica gi561959511* | FNI_iscf00014913.1.: 508.. 1794 |
| *F. nipponica gi561966037* | FNI_iscf00011472.1.: 1.. 1248 |
| *F. nipponica gi561975492* | FNI_iscf00006470.1.: 311... 1528 |
| *F. nipponica gi561978595.13* | FNI_iscf00004819.1.: 1221.. 2510 |
| *F. nipponica gi561982817.78* | FNI_iscf00002587.1.: 2405.. 3436 |
| *F. nipponica gi561986317* | FNI_iscf00000719.1.: 1768.. 3264 |

+ stop codons are found in the sequence;

{ gapes were introduced to avoid stop codons
